# A Pharmacy Liaison–Patient Navigation Intervention to Reduce Inpatient and Emergency Department Utilization Among Primary Care Patients in a Medicaid Accountable Care Organization: A Nonrandomized Controlled Trial

**DOI:** 10.1001/jamanetworkopen.2022.50004

**Published:** 2023-01-09

**Authors:** Pablo Buitron de la Vega, Erin M. Ashe, Ziming Xuan, Vi Gast, Tracey Saint-Phard, Julianna Brody-Fialkin, Felix Okonkwo, Julia Power, Na Wang, Chris Lyons, Michael Silverstein, Karen E. Lasser

**Affiliations:** 1 Department of Medicine, Boston Medical Center, Boston, Massachusetts; 2 Section of General Internal Medicine, Boston Medical Center, Boston, Massachusetts; 3 Boston University School of Medicine, Boston, Massachusetts; 4 Boston University School of Public Health, Boston, Massachusetts; 5 Takeda Pharmaceutical Company, Cambridge, Massachusetts; 6 AbbVie Bioresearch Center, Worcester, Massachusetts; 7 Action for Boston Community Development Inc, Boston, Massachusetts; 8 Brown University School of Public Health, Providence, Rhode Island

## Abstract

**Importance:**

Patients with unmet health-related social needs are at high risk for preventable health care utilization. Prior interventions to identify health-related social needs and provide navigation services with community resources have not taken place in pharmacy settings.

**Objective:**

To evaluate an enhancement of pharmacy care to reduce hospital admissions and emergency department (ED) visits among primary care patients in a Medicaid accountable care organization (ACO).

**Design, Setting, and Participants:**

This nonrandomized controlled trial was conducted from May 1, 2019, through March 4, 2021, with 1 year of follow-up. Study allocation was determined by odd or even medical record number. The study was performed at a general internal medicine practice at a large safety-net hospital in Boston, Massachusetts. Patients who qualified for the hospital’s pharmacy care program (aged 18-64 years and within the third to tenth percentile for health care utilization and cost among Medicaid ACO membership) who attended a visit with a primary care clinician were eligible. Of 770 eligible patients, 577 were approached, 127 declined, and 86 could not be contacted.

**Interventions:**

Patients in the control group received usual pharmacy care focused on medication adherence. Patients in the intervention group received enhanced pharmacy care with an additional focus on identification of and intervention for health-related social needs. The intervention took place for 1 year.

**Main Outcomes and Measures:**

The primary outcome was inpatient hospital admissions and ED visits (composite outcome) in the 12 months after enrollment during the intervention period.

**Results:**

Among 364 allocated patients (mean [SD] age, 50.1 [10.1] years; 216 women [59.3%]), 35 were Hispanic of any race (9.6%) and 214 were non-Hispanic Black (58.8%). All participants were included in the intention-to-treat analysis. In analyses controlling for baseline hospital admissions and ED visits the year prior to enrollment, the enhanced pharmacy care group was not associated with the odds of having any hospital admission or ED visit (adjusted odds ratio, 0.62 [95% CI, 0.23-1.62]; *P* = .32) among all patients and was not associated with the visit rates among those with any visit (adjusted rate ratio, 0.93 [95% CI, 0.71-1.22]; *P* = .62) relative to the usual pharmacy care group in the year following enrollment.

**Conclusions and Relevance:**

The findings of this nonrandomized controlled trial suggest that inpatient and ED utilization among Medicaid ACO members at a safety-net hospital was not significantly different between groups at 1-year follow-up.

**Trial Registration:**

ClinicalTrials.gov Identifier: NCT03919084

## Key Points

**Question:**

Is more frequent screening for health-related social needs and patient navigation embedded in pharmacy care associated with reduced hospital admissions and emergency department (ED) visits among primary care patients in a Medicaid accountable care organization when compared with usual pharmacy care?

**Findings:**

In this nonrandomized controlled trial of 364 adults, patients in the enhanced pharmacy care group did not have a lower likelihood of any hospital admissions or ED visits vs the usual pharmacy care group over 12 months.

**Meaning:**

These findings suggest that enhancing pharmacy services for patients with high levels of health care utilization does not lead to reduced health care utilization.

## Teaser

This nonrandomized controlled trial evaluates the association of an enhanced intervention for health-related social needs and navigation services with inpatient hospital admissions and emergency department (ED) visits among Medicaid recipients after 12 months.

## Introduction

Medicaid accountable care organizations (ACOs) assume responsibility for the health outcomes and costs of their members. Patients in low-resource communities served by these ACOs struggle with a number of health-related social needs, such as food insecurity, housing insecurity, and lack of transportation to medical appointments.^1,2^ Patients with unmet health-related social needs are at high risk for preventable health care utilization and high levels of medical expenditure^3^ and may prioritize social needs over medication adherence or receipt of medical care,^4^ which may lead to preventable utilization.

Prior studies^5^ have tested screening-and-referral interventions for health-related social needs to reduce health care utilization, with mixed results. Patient navigation interventions also connect patients to social services and community-based organizations to address unmet social needs and have focused on patients with high levels of medical utilization and complex care needs.^6^ A recent trial based in an urban emergency department (ED)^7^ found that patient navigation reduced ED visits and hospitalizations among Medicaid-insured patients. However, other studies^8,9^ have had mixed results. We are unaware of prior trials that have embedded a health-related social needs screening-and-referral intervention or a patient navigator in a pharmacy care program to reduce health care utilization. By embedding these interventions in a pharmacy program, we sought to address the root causes of medication nonadherence that may drive preventable utilization.

In this nonrandomized controlled trial^10^ involving patients who are members of a Medicaid ACO and receive primary care at a large urban safety-net hospital, our objective was to evaluate the effect of enhanced pharmacy care (a pharmacy technician with at least a high school degree serving as a patient navigator who screens for health-related social needs quarterly and provides navigation services to connect patients with community resources) in reducing inpatient hospital admissions and ED visits at 12 months. We hypothesized that the intervention would reduce health care utilization relative to usual pharmacy care.

## Methods

### Study Design

We conducted a parallel-group nonrandomized controlled trial with 1:1 allocation, whereby patients were assigned to 1 of 2 groups based on odd vs even medical record number. We did not randomize patients due to concerns it would disrupt patient flow. The study followed the Transparent Reporting of Evaluations With Nonrandomized Designs (TREND) reporting guideline. The study protocol found in Supplement 1 has been published elsewhere.^11^ The Boston Medical Center/Boston University Medical Campus Institutional Review Board approved the study protocol with a waiver of informed consent owing to the use of deidentified patient data.

### Setting and Participants

This study took place from May 1, 2019, through March 4, 2021, with 12-month follow-up within a pharmacy program embedded in a general internal medicine practice affiliated with a safety-net hospital in Boston, Massachusetts. Patients who qualified for the hospital’s pharmacy program were eligible for the study. Qualification for the pharmacy program required that patients be aged 18 to 64 years, within the third to tenth percentile on an internal risk score (primarily comprised of inpatient and ED utilization over the last 12 months combined with indices of medical and social risk) among Medicaid ACO membership at the time of program enrollment, and attend a visit with a primary care clinician (nurse practitioner or physician). The pharmacy program constituted the usual pharmacy care group described below.

### Recruitment

Recruitment took place from May 1, 2019, through March 5, 2020. As part of usual care, pharmacy liaisons received weekly reports of eligible patients scheduled with a primary care appointment scheduled to target for enrollment into the pharmacy program. Pharmacy liaisons approached patients for enrollment into the pharmacy program (and the study) prior to or following their primary care visit. Both usual pharmacy care liaisons spoke Spanish and one of the enhanced pharmacy care liaisons spoke Haitian Creole. All pharmacy liaisons (including V.G. and T.S.-P.) had access to interpreter services. Of 770 eligible patients, 577 were approached, 127 declined, and 86 could not be contacted.

### Allocation Procedure

We assigned patients to study condition based on medical record number. The usual pharmacy care liaisons approached and recruited patients with even medical record numbers, while the enhanced pharmacy care liaisons approached and recruited patients with odd medical record numbers. Intervention pharmacy liaisons did not provide details of their intervention during the recruiting process; patients were blinded to assigned study group.

### Intervention and Control Conditions

The intervention has been described previously by Lasser et al,^11^ and the trial protocol is found in Supplement 1. The usual pharmacy care group consisted of pharmacy liaisons focused on medication adherence. The enhanced pharmacy care group consisted of pharmacy liaisons dually trained as patient navigators who screened patients for health-related social needs quarterly, provided navigation services to connect patients with community resources, and carried a smaller caseload. We anticipated the enhanced focus on social needs would help promote medication adherence that would in turn lead to reduced health care utilization.

### Usual Pharmacy Care Group

The usual care group was standard of care in the practice, with some patients completing screening for health-related social needs during their primary care visit. While patients were initially screened for health-related social needs at every visit, due to patient screening fatigue, the clinic reduced the frequency of screening to every 6 months in June 2019.^12^ The screening tool included 8 domains of health-related social needs: housing insecurity, food insecurity, trouble paying for medications, trouble paying for transportation to medical appointments, trouble paying for utilities, need for employment, need for education, and difficulty taking care of children or other family members. At the end of the screening, a medical assistant asked patients if they wanted help connecting to resources. Patients who requested resources received printed information. Clinicians could also refer patients to a clinic-based patient navigator.

Patients who agreed to participate in the pharmacy program received a call from the pharmacy liaison to conduct an intake within 2 weeks of enrollment. The intake did not include screening for health-related social needs. During the intake call, the pharmacy liaison reviewed medication lists, assessed gaps in obtaining refills, identified barriers to medication adherence, reviewed the patient’s engagement in care, and developed an action plan. Action plans included strategies to increase medication adherence and engagement in care. After the intake, the pharmacy liaison called patients monthly over a 12-month period to confirm medication adherence and address new barriers to medication adherence and engagement in care. Pharmacy liaisons could review the most recent health-related social needs screening on an ad hoc basis. If the patient requested resources, the pharmacy liaison could connect the patient to the clinic-based navigator. Pharmacy liaisons in the usual care group worked with a maximum caseload of 215 patients, as they managed additional patients who were not part of the trial. Pharmacy liaisons in the usual care group spent 24 hours per week on case management; the remaining time was spent on other responsibilities.

### Enhanced Pharmacy Care Group

Patients assigned to this study group received the aforementioned services. Enhanced pharmacy care liaisons had smaller caseloads (maximum of 91 patients), had no other responsibilities, and received training to serve as patient navigators (termed *pharmacy liaison–patient navigators*). In this role, they connected patients with community and hospital resources to address health-related social needs. A dedicated 0.50 full-time equivalent employee at a community-based organization helped connect the patients receiving enhanced pharmacy care to services. The pharmacy liaison–patient navigators also received motivational interviewing training. A primary care physician with expertise in patient navigation (K.E.L.) and a social worker with expertise in motivational interviewing and in connecting patients with resources (J.B.-F.) met weekly with the liaisons to discuss challenging cases. The social worker also monitored pharmacy liaisons’ motivational interviewing skills using role-play. The pharmacy liaison–patient navigators spent the first 30 to 45 days after patient enrollment in the program helping patients overcome health-related social needs beyond those identified as barriers to medication adherence. If the pharmacy liaison–patient navigator documented during the intake assessment that the patient had received screening for health-related social needs in the past 3 months and rescreened the patient if they had not been screened in the past 3 months, we considered the patient to have received the minimum intervention dose. The pharmacy liaison–patient navigator used motivational interviewing skills to address domains in which a patient had positive screen results but did not indicate a desire to be connected to resources. The pharmacy liaison–patient navigator also provided patient education, assisted with scheduling appointments and reminders, and offered support to connect with internal and community resources to help patients mitigate health-related social needs.

### Outcomes

The primary outcome was all-cause inpatient hospital admissions and all-cause ED visits (a combination of any visit [binary outcome] and the count of visits among those patients with any visit) in the 12 months following enrollment. Secondary outcomes included all-cause inpatient hospital admissions and all-cause ED visits as separate outcomes, all-cause 30-day inpatient hospital readmissions, 30-day ED revisits, and social needs outcomes (eg, connected to community-based organization, received services through referral to community-based organization, referred to hospital food pantry, or received utility shutoff protection letter). The data analyst (N.W.) was blinded to participant group assignment.

### Covariates and Confounders

We collected demographic data and potential confounders such as medical comorbidity (Charlson Comorbidity Index score),^13^ mental health and substance use diagnoses, and screening results. Race and ethnicity data were collected in the routine course of clinical care. While some participants may have identified their race or ethnicity, in some cases front desk staff may have provided the identification. Both methods used the same racial and ethnic categories for all patients treated at the hospital. Data are not available on the proportion of patients whose race and ethnicity were self-reported vs determined by front desk staff. Options were defined by the health system. We analyzed these data to capture unmeasured social factors (eg, structural racism, racial discrimination) and to identify disparities in health care utilization. Data came from the health system’s electronic health records and insurance claims.

### Statistical Analysis

Using a 2-sided type I error rate of 0.05, a sample size of 364 patients (182 in each group) would achieve 90% power to detect a 10–percentage point reduction in our primary outcome. We analyzed all outcomes according to the intention-to-treat principle.^14^ Due to a high number of patients with zero-count visits (136 of 364), we used a zero-inflated negative binomial regression, a 2-part model that simultaneously examined the odds of any visit and visit rates among patients who had any health care utilization. We controlled for potential confounders identified in bivariate analyses as well as variables of a priori clinical significance. We also analyzed the intervention effect according to whether participants received the minimum intervention dose and performed post hoc analyses examining the primary outcome among patients who had continuous ACO enrollment during the follow-up period. We compared secondary outcomes of social needs between the 2 groups and examined the adjusted intervention effect using log binomial regression with adjusted risk ratios (aRRs) and 95% CIs. All analyses were conducted using SAS, version 9.4 (SAS Institute Inc). Two-sided *P* < .05 indicated statistical significance.

## Results

Of 2097 potentially eligible patients, we assigned 364 (17.4%) to study condition. The Figure shows reasons for exclusion, assignment to study condition, and follow-up; Table 1 shows descriptive characteristics for the usual pharmacy care and enhanced pharmacy care groups. The mean (SD) age of all participants was 50.1 (10.1) years. Two hundred sixteen participants were women (59.3%) and 148 were men (40.7%). Two hundred ninety-six participants (81.3%) were members of a racial or ethnic minority group (35 Hispanic [9.6%], 214 non-Hispanic Black [58.8%], and 47 other [Asian, American Indian or Alaska Native, Native Hawaiian or other Pacific Islander, other, or declined or not available; 12.9%]). More than one-half of patients had significant medical comorbidity, and nearly one-half carried a diagnosis of depression. The usual care and enhanced care groups differed in the proportions who spoke English (142 [78.0%] vs 158 [86.8%], respectively), reported housing insecurity (17 [9.3%] vs 23 [12.6%], respectively), received a hospital food pantry referral in the past 12 months (25 [13.7%] vs 45 [24.7%], respectively), had at least 1 visit to the hospital food pantry in the past 12 months (22 [12.1%] vs 36 [19.8%], respectively), and had a posttraumatic stress disorder diagnosis (39 [21.4%] vs 26 [14.3%], respectively). In the enhanced pharmacy care group, 105 participants (57.7%) received the minimum intervention dose.

**Figure.**
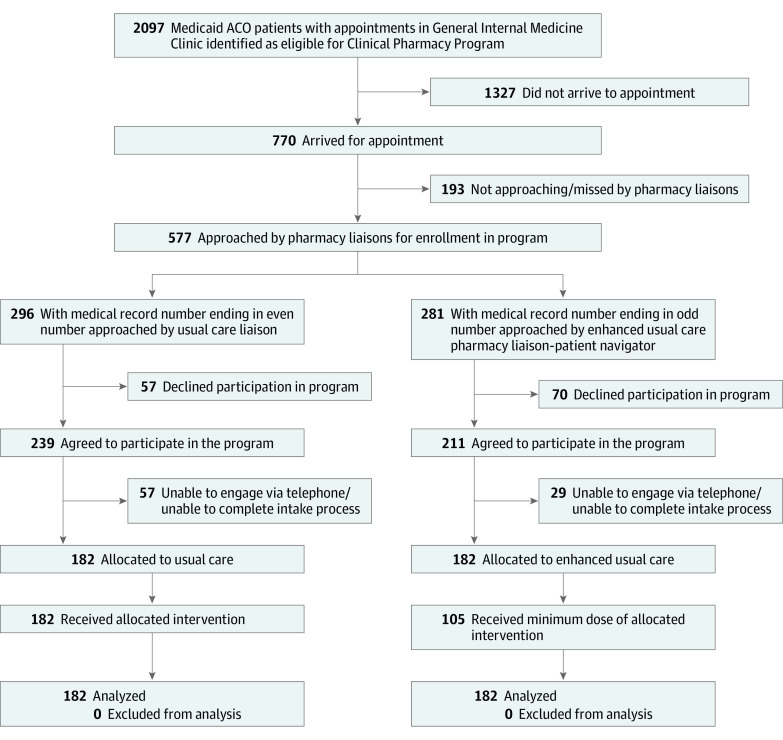
Study Flow Diagram A total of 364 participants were allocated for analysis. ACO indicates accountable care organization.

**Table 1.** Baseline Characteristics of Participants by Treatment Group^a^

Characteristic	Participant group^b^			*P* value^c^
	All (N = 364)	Usual pharmacy care (n = 182)	Enhanced pharmacy care (n = 182)	
Sociodemographic				
Age at enrollment, mean (SD), y	50.1 (10.1)	50.7 (9.8)	49.4 (10.4)	.25
Sex				
Women	216 (59.3)	112 (61.5)	104 (57.1)	.39
Men	148 (40.7)	70 (38.5)	78 (42.9)	
Race and ethnicity^d^				
Hispanic (any race)	35 (9.6)	17 (9.3)	18 (9.9)	.95
Non-Hispanic Black	214 (58.8)	109 (59.9)	105 (57.7)	
Non-Hispanic White	68 (18.7)	32 (17.6)	36 (19.8)	
Other^e^	47 (12.9)	24 (13.2)	23 (12.6)	
Language				
English	300 (82.4)	142 (78.0)	158 (86.8)	.03^f^
Spanish	36 (9.9)	22 (12.1)	14 (7.7)	
Haitian Creole	9 (2.5)	4 (2.2)	5 (2.7)	
Other	19 (5.2)	14 (7.7)	5 (2.7)	
Health-related social needs				
Screened in 12 mo prior to study enrollment	351 (96.4)	175 (96.2)	176 (96.7)	.78
Health-related social needs screening results^g^				
Housing insecurity	40 (11.0)	17 (9.3)	23 (12.6)	.06
Food insecurity	36 (9.9)	16 (8.8)	20 (11.0)	.34
Trouble paying for medication	20 (5.5)	12 (6.6)	8 (4.4)	.34
Trouble getting transportation to medical appointments	26 (7.1)	17 (9.3)	9 (4.9)	.18
Trouble paying for utilities	19 (5.2)	10 (5.5)	9 (4.9)	.56
Trouble taking care of child or family member	4 (1.1)	3 (1.6)	1 (0.5)	.26
Unemployed, looking for job	19 (5.2)	12 (6.6)	7 (3.8)	.31
Interested in more education	21 (5.8)	11 (6.0)	10 (5.5)	.67
Received utility shutoff protection letter in past 12 mo	22 (6.0)	13 (7.1)	9 (4.9)	.38
Referred to hospital food pantry in past 12 mo	70 (19.2)	25 (13.7)	45 (24.7)	.008
Had ≥1 visit to hospital food pantry in past 12 mo	58 (15.9)	22 (12.1)	36 (19.8)	.045
Medical and behavioral health comorbidity^h^				
Significant medical comorbidity (Charlson Comorbidity Index score ≥1)	243 (66.8)	128 (70.3)	115 (63.2)	.15
Medical diagnoses				
COPD	95 (26.1)	50 (27.5)	45 (24.7)	.55
Diabetes (type 1 or 2)	107 (29.4)	58 (31.9)	49 (26.9)	.30
Behavioral health comorbidity				
Alcohol use disorder or alcohol-induced mental disorders	59 (16.2)	25 (13.7)	34 (18.7)	.20
Drug use disorder or drug-induced mental disorders	97 (26.6)	48 (26.4)	49 (26.9)	.91
Depression	172 (47.3)	92 (50.5)	80 (44.0)	.21
Anxiety	94 (25.8)	44 (24.2)	50 (27.5)	.47
Posttraumatic stress disorder	65 (17.9)	39 (21.4)	26 (14.3)	.08
Medicaid ACO insurance coverage proportion in 12 mo prior to enrollment, mean (SD)	0.80 (0.34)	0.78 (0.36)	0.82 (0.32)	.32

Abbreviations: ACO, accountable care organization; COPD, chronic obstructive pulmonary disease.

^a^ Study enrollment period was May 28, 2019, through March 5, 2020; all participants were enrolled prior to hospital-instated COVID-19 pandemic restrictions.

^b^ Unless otherwise indicated, data are expressed as No. (%) of participants. Percentages have been rounded and may not total 100.

^c^ Calculated via *t* test.

^d^ Collected in the routine course of clinical care through the patient portal, registration, and telephone encounters.

^e^ Includes Asian, American Indian or Alaska Native, Native Hawaiian or other Pacific Islander, other, and declined or not available.

^f^ Compared between English vs non-English language.

^g^ Indicates health-related social needs screening result on most recent screen prior to or on the date of enrollment.

^h^ Based on *International Statistical Classification of Diseases and Related Health Problems, Tenth Revision* codes on the electronic health record problem list and used for billing.

Inpatient hospital admissions and ED visits decreased in both groups over the study period. In the usual care group, 129 (70.9%) had at least 1 inpatient hospital admission or ED visit compared with 118 (64.8%) in the 12-month follow up period. In the enhanced pharmacy care group, the corresponding numbers were 136 (74.7%) and 110 (60.4%). In analyses controlling for language, housing insecurity, referral to food pantry, visit to food pantry, posttraumatic stress disorder, and baseline hospital admissions and ED visits (in the year prior to enrollment), the enhanced pharmacy care group was not associated with the odds of having any hospital admissions or ED visits relative to the usual pharmacy care group (adjusted odds ratio, 0.62 [95% CI, 0.23-1.62]; *P* = .32). Similarly, there was no between-group difference in the rates of visits among those with any visit in the follow-up period in the adjusted model (aRR, 0.93 [95% CI, 0.71-1.22]; *P* = .62) (Table 2). Due to small numbers of patients with any hospital admission or ED visits, we did not run interaction terms between comorbid mental health and substance use diagnoses, language, and intervention group. No significant associations were detected in analyses restricted to patients who had continuous ACO enrollment during the follow-up period (eTable 1 in Supplement 2). Similarly, outcomes did not differ according to receipt of the minimum intervention dose among members of the enhanced pharmacy care group (eTable 2 in Supplement 2).

**Table 2.** Health Care Utilization at Baseline and 12 Months After Enrollment by Study Group

Variable	Baseline				Follow-up				Adjusted zero-inflated negative binomial model^a^			
	Enhanced pharmacy care (n = 182)		Usual pharmacy care (n = 182)		Enhanced pharmacy care (n = 182)		Usual pharmacy care (n = 182)		Zero-inflated model		Negative binomial model	
	Patients with any hospital admission or ED visit, No. (%)	No. of hospital admissions or ED visits among patients with any visit, mean (SD)	Patients with any hospital admission or ED visit, No. (%)	No. of hospital admissions or ED visits among patients with any visit, mean (SD)	Patients with any hospital admission or ED visit, No. (%)	No. of hospital admissions or ED visits among patients with any visit, mean (SD)	Patients with any hospital admission or ED visit, No. (%)	No. of hospital admissions or ED visits among patients with any visit, mean (SD)	Odds ratio (95% CI)	*P* value	IRR (95% CI)	*P* value
All-cause inpatient hospital admissions and all-cause ED visits (a composite outcome) within the past year	136 (74.7)	5.8 (7.4)	129 (70.9)	4.8 (5.3)	110 (60.4)	4.3 (4.3)	118 (64.8)	3.9 (3.6)	0.62 (0.23-1.62)	.32	0.93 (0.71-1.22)	.62
All-cause ED visits within the past year	131 (72.0)	4.8 (6.2)	123 (67.6)	3.9 (4.4)	106 (58.2)	3.6 (3.5)	115 (63.2)	3.3 (3.0)	0.61 (0.23-1.62)	.32	0.90 (0.69-1.19)	.48
30-d ED revisits within the past year	84 (46.2)	4.0 (6.8)	56 (30.8)	3.9 (4.8)	56 (30.8)	3.2 (3.3)	57 (31.3)	2.9 (2.8)	0.85 (0.28-2.59)	.77	0.89 (0.52-1.53)	.68
All-cause inpatient hospital admissions within the past year	60 (33.0)	2.7 (1.9)	56 (30.8)	2.5 (2.0)	41 (22.5)	2.2 (1.7)	40 (22.0)	1.9 (1.1)	0.82 (0.41-1.64)	.57	1.31 (0.85-2.00)	.22
All-cause 30-d inpatient hospital readmissions within the past year^b^	38 (20.9)	1.9 (1.5)	31 (17.0)	2.0 (1.5)	17 (9.3)	2.1 (1.7)	15 (8.2)	1.4 (0.5)	0.18 (0.03-1.10)	.06	4.43 (1.97-9.95)	.0003

Abbreviations: ED, emergency department; IRR, incidence rate ratio.

^a^ Zero-inflated negative binomial model adjusted for English, baseline housing insecurity, posttraumatic stress disorder, referral to food pantry, visit to food pantry, and baseline outcome.

^b^ The validity of the model fit is questionable in adjusted model due to a convergence problem.

Nineteen enhanced pharmacy care group participants (10.4%) received services at the partner community-based organization in the 12 months following enrollment compared with 17 (9.3%) of usual pharmacy care group participants (aRR, 1.04 [95% CI, 0.55-1.98]; *P* = .91). Twenty-six enhanced pharmacy care group participants (14.3%) had at least 1 visit to the food pantry compared with 16 usual pharmacy care group participants (8.8%) (aRR, 1.18 [95% CI, 0.70-1.99]; *P* = .53). Similarly, there was no between-group difference in receiving at least 1 utility shut-off protection letter issued by a clinician (10 [5.5%] for enhanced pharmacy care vs 8 [4.4%] for usual pharmacy care; aRR, 1.78 [95% CI, 0.67-4.73]; *P* = .25).

## Discussion

Medicaid ACO members receiving primary care at a large urban safety-net hospital assigned to receive care from pharmacy liaisons with training in motivational interviewing and patient navigation and who carried smaller caseloads had no difference in inpatient hospital admissions and ED visits at 12 months relative to patients who received usual care. Both groups experienced decreased health care utilization during the COVID-19 pandemic, which is consistent with prior studies.^15^ Our study adds to a growing literature finding that pharmacist interventions in primary care^16^ and patient navigation–like interventions do not reduce utilization among safety-net patient populations. The most notable of the latter studies was the Health Care Hotspotting trial,^6^ which found readmission rates were not lower among patients randomly assigned to the Camden Coalition’s care-transition program than among those who received usual care. It is possible, however, that certain groups (eg, older patients^9^ and those with limited English proficiency^17^) may benefit from patient navigation interventions. Patient navigation in the ED setting also shows promise.^7^

The dually trained pharmacy liaisons in our study provided services similar to those used in other navigation programs, such as connecting patients to internal and community resources^8,18^ to mitigate health-related social needs and coordinating medical care.^8,18,19^ As in prior studies, the navigators used motivational interviewing to promote behavior change.^17,20^ Aspects of the intervention differing from prior studies included enhanced training in screening for health-related social needs, partnership with a community-based organization, and incorporation of pharmacy liaison services. There are several reasons why we did not observe a difference in utilization outcomes at the end of the trial. First, patients in the usual care group also received a moderately intensive intervention. Like participants in the intervention group, they received monthly phone calls from a pharmacy liaison who could assist with barriers to medication adherence. Additionally, patients in the usual care study group were screened in a primary care practice for health-related social needs, and these patients were able to work with a clinic-based patient navigator to address such needs. Had our usual care study group been more reflective of usual care at other safety-net primary care practices, we might have observed an intervention effect. Second, only 57.7% of patients in the enhanced care intervention received the minimum intervention dose. With the exception of a face-to-face encounter for recruitment, the pharmacy liaison–patient navigators delivered the intervention entirely via telephone. We suspect that patients with unstable housing and limited telephone service were unable to engage with the intervention.

Interventions in health care settings are unlikely to improve health outcomes rooted in long-standing and intractable structural racism. A 2015 study^21^ found the median net worth for White households in Greater Boston was a quarter million dollars, while for Black families, it was substantially lower. Given that the majority of study participants identified as members of a racial or ethnic group bearing a disproportionate burden of inequities in health care, it is unsurprising that a health system–based intervention did not improve outcomes. Implementation of policies to reduce income inequality would be more likely to improve health outcomes.

### Limitations

This study had several limitations. First, it was not randomized. It is possible that there were unmeasured differences in the 2 study groups that we did not control for in our analysis. Considering that only 17.4% of eligible ACO patients participated in the trial, our findings might not be generalizable to all patients with high utilization and high cost from ACOs in safety-net institutions. Our sensitivity analyses and post hoc subgroup analyses did not have enough sample size to achieve statistical power. Data on patient race and ethnicity were collected in the routine course of clinical care via a combination of self-report and determination by hospital staff and therefore may be inaccurate. Finally, usual care in our study included a pharmacy liaison and a clinic-based navigator. Such staff may not be available in most primary care settings, thereby limiting generalizability of our findings. However, our intervention may be effective when implemented in clinics with fewer ancillary staff.

## Conclusions

The findings of this nonrandomized controlled trial suggest that an intervention where Medicaid ACO members at a safety-net hospital received care from pharmacy liaison–patient navigators was not associated with a reduction in inpatient hospital admissions and ED visits relative to usual pharmacy care (pharmacy liaison and clinic-based navigator). Future studies might focus on preventing high levels of health care utilization and should identify which patients may benefit most from patient navigation to reduce health care utilization and in which settings such interventions should be delivered.

## Notes

Supplement 1.
Trial Protocol and Statistical Analysis Plan

Supplement 2. **eTable 1.** Analyses Restricted to Patients With Continuous ACO Enrollment During the Follow-up Period **eTable 2.** Analyses Comparing Patients in the Enhanced Pharmacy Care Group According to Receipt of the Minimum Intervention Dose

Supplement 3.
Data Sharing Statement
