# Supplementary material for: A Pharmacy Liaison–Patient Navigation Intervention to Reduce Inpatient and Emergency Department Utilization Among Primary Care Patients in a Medicaid Accountable Care Organization: A Nonrandomized Controlled Trial
Source: JAMA Netw Open. 2023 Jan 9;6(1):e2250004. doi: 10.1001/jamanetworkopen.2022.50004 (PMC9856667; doi:10.1001/jamanetworkopen.2022.50004)
Supplement: Supplement 1. — Trial Protocol and Statistical Analysis Plan [file jamanetwopen-e2250004-s001.pdf]

A Pharmacy Liaison-Patient Navigation Intervention to Reduce Inpatient and Emergency Department Utilization among Primary Care Patients in a Medicaid Accountable Care Organization: A Pragmatic Trial

ClinicalTrials.gov number: NCT03919084
Protocol Version Number: 1.3
Protocol Version Date: 5 August, 2021
Funding Mechanism: Massachusetts Health Policy Commission, BMC AU# 6005732
Principal Investigator: Karen Lasser, MD, MPH
Phone: 617 414 6688
E-mail: Karen.Lasser@bmc.org

CONFIDENTIAL

This document is confidential and the property of Boston Medical Center. No part of it may be transmitted, reproduced, published, or used by other persons without prior written authorization from the study sponsor.

TABLE OF CONTENTS

1 List of Abbreviations 3
2 Protocol Summary 4
3 Background/Rationale & Purpose 6
3.1 Background Information 6
3.2 Rationale and Purpose 7
4 Objectives 9
4.1 Study Objectives 9
4.2 Study Outcome Measures 9
4.2.1 Primary Outcomeun Measures 9
4.2.2 Secondary Outcome Measures 10
5 Study Design 10
6 Potential Risks and Benefits 11
6.1 Risks 11
6.2 Potential Benefits 12
6.3 Analysis of Risks in Relation to Benefits 12
7 Study Subject Selection 13
7.1 Subject Inclusion Criteria 13
7.2 Subject Exclusion Criteria 13
8 Study Intervention 13
9 Study Procedures 16
10 Assessment of Safety and Data Safety Monitoring Plan (DSMP) 21
11 Data Handling and Record Keeping 21
11.1 Confidentiality 21
11.2 Source Documents 22
11.3 Case Report Forms 22
11.4 Study Records Retention 23
12 Statistical Plan 23
12.1 Study Hypotheses 23
12.2 Sample Size Determination 23
12.3 Statistical Methods 24
13 Ethics/Protection of Human Subjects 24
14 Literature References 26
15 Appendix 27

1 List of Abbreviations

Abbreviation	Abbreviation definition
ABCD	Action for Boston Community Development
ACO	Accountable Care Organization
AE	Adverse Event
BACO	Boston Accountable Care Organization
BMC	Boston Medical Center
CRF	Case Report Form
ED	Emergency Department
EHR	Electronic Health Record
GIM	General Internal Medicine
HRSN	Health-Related Social Needs
IP	In-Patient
MI	Motivational interviewing, a counseling method that encourages patient-centered discussions. MI will be delivered by the intervention arm pharmacy liaison-patient navigators to identify the patient's unmet needs and encourage the patient to adopt behavior change that will promote engagement with resources and services to mitigate or alleviate HRSN.
PDC	Proportion of Days Covered

Term	Term definition
Adverse Event (AE)	For the purposes of this study, defined as any unanticipated problem or issue regarding confidentiality.
Clinical staff	Collectively refers to both the two pharmacy liaisons and two pharmacy-liaison patient navigators in the usual care control arm and the intervention arm, respectively.
Pharmacy Care Program	An existing BMC clinical program in General Internal Medicine that deploys pharmacy liaisons to assist patients with prescription access, medication adherence, and ambulatory care engagement. Part of usual care clinical activities in GIM, not the research study.
Pharmacy Liaison	Clinical staff person registered as a Pharmacy Technician with the Board of Pharmacy in Massachusetts and active National Certification verified by PTCB or ExCPT. Provides standard of care services through the current Pharmacy Care Program in General Internal Medicine (GIM) at Boston Medical Center.
Pharmacy Liaison-Patient Navigator	A pharmacy liaison trained as a patient navigator to deploy both Pharmacy Care Program services and the THRIVE+ intervention.
The Clinical Program	The Pharmacy Care Program
Usual care control	Patients receiving usual care in GIM will serve as the control arm (n=182) for our study. The usual care model in GIM includes:<br>1. THRIVE-Basic screening for HRSN and providing tailored printed referral guides that direct patients to specific hospital and community-based resources; and<br>2. Pharmacy Care Program services to promote medication adherence, delivered by a pharmacy liaison.

2 Protocol Summary

**Title:**	A Pharmacy Liaison-Patient Navigation Intervention to Reduce Inpatient and Emergency Department Utilization among Primary Care Patients in a Medicaid Accountable Care Organization: A Pragmatic Trial
**Population:**	364 Boston Accountable Care Organization (BACO) members 18-64 years whose healthcare utilization and cost ranks in the top 3-10% of the ACO membership, and who receive primary care in the general internal medicine practice at Boston Medical Center. This group of "intermediate risk" patients will be identified with a statistical algorithm that considers past utilization patterns, selected diagnoses, and other factors to assess risk of service utilization.
**Intervention:**	Arm #1: THRIVE-Basic usual care screening and referral program, delivered within the context of an existing pharmacy services program.<br>Arm #2: THRIVE+, which enhances the THRIVE-Basic screening and referral with targeted navigation services provided by a pharmacy liaison-patient navigator working in partnership with a community partner organization. THRIVE+ patient navigators will deliver both the THRIVE+ intervention and the pharmacy care program to avoid duplication of services and multiple touches by multiple intervention providers.
**Objectives:**	1. <u>Effectiveness</u>: Evaluate whether THRIVE+ demonstrates superior results in reduction of acute healthcare utilization compared to THRIVE-Basic usual care program.<br>2. <u>Budget impact</u>: Assess whether the THRIVE+ model reduces net costs by reducing acute health care utilization.<br>3. <u>Implementation</u>: Determine if THRIVE+ enhanced patient navigation component results in identifying and addressing a greater number of HRSN compared to the patient-reported HRSN on the THRIVE screening tool.
**Design/Methodology:**	We are conducting a pragmatic comparative effectiveness trial (n=364) to compare two screening and referral program models to address HRSN among the intermediate risk population of BACO membership (top 3-10% of the ACO membership utilization). The first study arm is THRIVE-Basic, the low-touch usual care model already implemented in all primary care clinics at BMC where patients are screened for HRSN and receive a printed paper resource referral sheet. The second study arm is THRIVE+, which enhances the THRIVE-Basic model by engaging a pharmacy liaison-patient navigator to provide targeted navigation services and motivational interviewing to ensure connection to hospital- and community-based resources. The patient navigators will also interface directly with a partner community organization, Action for

	Boston Community Development (ABCD), to further help connect patients receiving THRIVE+ to community resources for HRSN. All patients in our study will receive pharmacy services. Patients in study arm 1 will be connected to a pharmacy liaison, which is standard clinical practice for intermediate risk ACO members. Patients in study arm 2 will receive systematic screening for and addressing of HRSN (THRIVE+) via a pharmacy liaison-patient navigator (a pharmacy technician trained as a patient navigator to deploy both pharmacy services and the THRIVE+ intervention), thereby avoiding duplication of services and multiple touches. At its core, this study is assessing whether streamlining service delivery (i.e., having the intervention arm pharmacy liaison-patient navigator as a single individual that deploys both pharmacy services and patient navigation services as well as systematically screens for HRSN) is superior to multiple touches by separate, and often disparate, resources (i.e., a pharmacy liaison and a separate clinic-embedded patient navigator each delivering different services and not systematically screening for HRSN).<br><br>Assignment to the study arms will be linked to existing Pharmacy Care Program enrollment activities. Clinical staff (the two pharmacy liaisons for the THRIVE-Basic study arm and the two pharmacy liaisons trained as patient navigators for the THRIVE+ study arm) will approach eligible patients prior to or following their primary care visit, as is current practice for enrollment into the clinical Pharmacy Care Program To ensure equivalence across treatment conditions, each liaison pair (2 pairs; 4 liaisons total) will target the primary care suites with the highest numbers of eligible patients, on the same day and time. The control pharmacy liaison will approach patients whose medical record number ends in an even number, while the intervention pharmacy liaison will approach patients whose medical record number ends in an odd number.<br><br>We will compare the effectiveness of these two models in alleviating HRSN and reducing acute health care utilization over a 12-month follow-up period.
**Consent:**	We are requesting a waiver of informed consent and a waiver of HIPAA authorization for all participants in both study arms.
**Total Study Duration:**	April 2019 – June 2021
**Subject Participation Duration:**	12 months

59

3 Background/Rationale & Purpose

3.1 Background Information

Addressing the health-related social needs (HRSN) of patients is critical to providing high-quality care at the lowest possible cost. This is particularly significant for low-income populations like the ones cared for at Boston Medical Center (BMC) and throughout the BACO. Although social determinants of health have been an important topic in public health spheres for decades,[1] specifically addressing them as part of the health care delivery system is a relatively new addition to the health services literature.[2,3] Given evidence that suggests patients in low-resource communities struggle with a number of HRSN, the Centers for Medicaid and Medicare Services and several professional societies have strongly recommended the use of social determinants of health screening tools at health supervision (or preventive care) visits.[4] As a result, health systems have adopted and payers, including Medicaid-based managed care organizations in thirty states, have recommended various screening tools – which are linked to a variety of intervention strategies.[5]

While there is some evidence to support the effectiveness of interventions in addressing patients' social and economic needs, prior studies have been limited by non-experimental project designs, and assessments that focus on process measures, rather than health outcomes, heath care utilization, and cost.[6] One randomized controlled trial found that systematically screening and referring for social determinants during well child care visits led to the receipt of more community resources among families with unmet HRSN.[7] BMC has adapted the screening tool used in that trial and implemented the tool (the "THRIVE" screener) into routine primary care visits. In our comparative effectiveness study, we refer to this low touch, usual care intervention as THRIVE-Basic.

The THRIVE-Basic screening tool consists of questions designed to identify eight potentially unmet HRSN directly associated with health outcomes and healthcare utilization: homelessness, housing insecurity, food insecurity, inability to afford medications, access to transportation for medical appointments, utilities, childcare, care for an elderly or disabled person, employment, and educational needs. These domains have the highest impact on overall patient health, according to the National Academy of Medicine.[8] The screening tool is written at a 3rd grade reading level, is available in multiple languages, and takes less than five minutes to complete. Once the screening is complete, results are entered into the patients' electronic health record. Patients who identify social needs are provided one or more printed paper resource referral guides that describe available hospital and community resources to address their HRSN. However, it is unknown whether screening for social determinants alone, without providing additional support, is sufficient to address the HRSN of adult patients with higher medical complexity and more intensive acute care utilization patterns.

To address these concerns, we have designed a more intensive intervention (THRIVE+), which includes a patient navigation intervention component. In traditional patient navigation programs, a lay person from the community guides individuals through the healthcare system to receive appropriate services. Navigation has been used successfully to improve health outcomes in cancer prevention as well as in cancer and mental health care.[9-12] For the present study, we will train pharmacy liaisons (pharmacy technicians with at least a high school degree and four years of pharmacy experience) to provide patient navigation services, in addition to providing medication adherence support and assistance resolving

barriers to accessing medication. In an effort to avoid duplication of services we chose to deliver the patient navigation intervention via a pharmacy liaison trained in patient navigation, thereby increasing the potential for sustainability should the intervention prove effective. This specialized pharmacy liaison job description includes as one job function "addressing HRSN by connecting patients to resources available within BMC and within the community."

The primary aim of this study is to determine whether screening for social determinants of health and providing a tailored set of printed referral guides that direct patients to specific hospital and community-based resources and employing the services of a pharmacy liaison to promote medication adherence ("THRIVE-Basic"), relative to adding to THRIVE-Basic the assistance of a pharmacy liaison with training as a patient navigator who provides targeted navigation services aimed at connecting patients with appropriate community resources (THRIVE+), will reduce hospital admissions and emergency department visits among patients who are members of a Medicaid accountable care organization and receive primary care at a large urban safety-net hospital.

We are conducting a pragmatic comparative effectiveness trial to concurrently compare two screening and referral program models to address HRSN among the intermediate risk population of BACO membership (top 3-10% of the ACO membership utilization). Both study arms share the same universal screening strategy using the THRIVE-Basic screening tool as described above, and both study arms provide printed resource referral guides with contact information for local community and hospital resources to patients who screen positive for HRSN; however, they differ in intensity of supports received to access resources to address identified HRSN post-screening. The first study arm, THRIVE-Basic, the usual care model, concludes with the patients' receipt of the printed resources referral guides and possible referral to a clinic-based navigator. The second study arm, THRIVE+, enhances the THRIVE-Basic program by incorporating targeted navigation services provided by a pharmacy liaison trained as a patient navigator, in partnership with a community organization, to facilitate access to resources that address HRSN. At its core, this study is assessing whether streamlining service delivery (i.e., having the intervention arm pharmacy liaison-patient navigator as a single individual that deploys both pharmacy services and patient navigation services as well as systematically screens for HRSN) is superior to multiple touches by separate, and often disparate, resources (i.e., a pharmacy liaison and a separate clinic-embedded patient navigator each delivering different services not systematically linked to screening for HRSN).

We will track acute healthcare utilization (in-patient and emergency room); number of HRSN identified and addressed; and patient satisfaction over 12 months of follow-up.

This study will be conducted in compliance with the protocol, applicable regulatory requirements, and BMC/BU Medical Campus Human Research Protection policies and procedures.

3.2 Rationale and Purpose

While there is some evidence to support the effectiveness of social determinants of health screening-and-referral interventions in addressing patients' social and economic needs, the literature is limited by low-quality studies; non-experimental project designs; and assessments that focus on process, as opposed to ones that measure health outcomes, heath care utilization, and cost.[6] One randomized

controlled trial found that systematically screening and referring for social determinants during well child care visits led to the receipt of more community resources for families, however receipt of such services was overwhelmingly low – even in the intervention group.[7] For example, only 15% of families were able to access affordable childcare; 11%, food assistance programs; and 8% employment or job training programs.

It is unknown whether screening for social determinants alone, without providing additional support, is sufficient to address the HRSN of adult patients with higher medical complexity and more intensive acute care utilization patterns. Further, many of these patients also face barriers to accessing services to address their HRSN. Some of the most common barriers to accessing healthcare services are financial problems, employment issues, child/adult care, and housing insecurity.[13] Barriers to accessing social services are similar to barriers accessing health services and include lack of transportation, eligibility requirements, low literacy, limited English proficiency, and different cultural experiences and expectations.[14] High utilizers, in particular, experience poor coordination across medical, behavioral, and social services providers.[13]

Although THRIVE-Basic is an evidence-based screening-and-referral model, implementing universal THRIVE at BMC has demonstrated that providing resource referral guides is necessary, but insufficient, for patients with higher medical complexity and more intensive acute care utilization patterns, many of whom also face barriers in accessing services for HRSN. As the THRIVE-Basic model of care has been implemented in several BMC clinics, we have a strong picture of the burden of HRSN for intermediate risk BACO members receiving care at BMC. Our preliminary data demonstrate that intermediate risk adult BACO patients have a disproportionate burden of HRSN. In addition, we know this population requires help to solve barriers to accessing both health services and social services. This represents convincing evidence that a more robust model – our proposed THRIVE+ patient navigation services model – is necessary to increase connection to, and receipt of, these important resources to reduce the burden of HRSN, particularly among intermediate risk BACO patients who experience disproportionate HRSN.

Patient navigation has been identified as an effective intervention to decrease barriers to access healthcare services. Patient navigators perform different tasks to help patients access health care, including providing logistic support for barriers, emotional support, interpreter services, appointment scheduling, appointment reminders, information and answering questions regarding care, connections to support services, and guidance or accompaniment of patients to appointments.[15]

To address these barriers, we have developed the THRIVE+ intervention, the underlying rationale of which is based on the tenets of patient navigation. Patient navigation is based on the principle that reducing logistic and psychological barriers to services is an efficient and effective mechanism to increase engagement with services.[16] Navigation has been used successfully in domains of cancer and mental health care;[9,12,17-19] we believe that navigation services – conducted by pharmacy liaisons trained as patient navigators – may be a cost-effective strategy to help intermediate-risk patients' access community services that address HRSN.

Our study will compare the effectiveness of THRIVE-Basic with a more robust model, THRIVE+, the intensity of which is matched to the HRSN-burden and healthcare utilization patterns of an intermediate risk group of BACO members. THRIVE+ includes a patient navigation intervention component. In traditional patient navigation programs, a lay person from the community guides individuals through the healthcare system to receive appropriate services. Navigation has been used successfully to improve

health outcomes in cancer prevention as well as in cancer and mental health care.[9-12] For the present study, we will train pharmacy liaisons (pharmacy technicians or pharmacy interns with at least a high school degree and four years of pharmacy experience) to provide patient navigation services, in addition to providing medication adherence support and assistance resolving barriers to accessing medication via the Pharmacy Care Program. In an effort to avoid duplication of services we chose to deliver the patient navigation intervention via a pharmacy liaison trained in patient navigation, thereby increasing the potential for sustainability should the intervention prove effective. This specialized pharmacy liaison job description includes as one job function "addressing HRSN by connecting patients to resources available within BMC and within the community."

The primary aim of this study is to determine whether screening for HRSN and providing tailored printed referral guides that direct patients to specific hospital and community-based resources and employing the services of a pharmacy liaison to promote medication adherence ("THRIVE-Basic"), relative to adding to THRIVE-Basic the assistance of a pharmacy liaison with training as a patient navigator who provides targeted navigation services aimed at connecting patients with appropriate community resources (THRIVE+), will reduce hospital admissions and emergency department visits among patients who are members of a Medicaid accountable care organization and receive primary care at a large urban safety-net hospital.

4 Objectives

4.1 Study Objectives

Our study objectives are as follows:

1. Effectiveness: Evaluate whether THRIVE+ demonstrates superior results in reduction of acute healthcare utilization compared to THRIVE-Basic usual care program.
2. Budget impact: Assess whether the THRIVE+ model reduces net costs by reducing acute health care utilization.
3. Implementation: Determine if THRIVE+ enhanced patient navigation component results in identifying and addressing a greater number of HRSN compared to the patient-reported HRSN on the THRIVE screening tool.

We will pursue a fourth study objective, patient experience and satisfaction, measured via client satisfaction questionnaires (CSQ-8). This will be detailed in a separate Exempt Category 2 IRB submission.

4.2 Study Outcome Measures

4.2.1 Primary Outcome Measures

Our primary outcome measure is a composite measure of hospital admissions and ED visits at 12 months.

Each subject will have 12-months of follow-up. To satisfy requirements of our funder, we will report the primary and secondary outcomes for only the THRIVE+ intervention arm participants on a monthly basis.

4.2.2 Secondary Outcome Measures

Secondary outcomes measures will be measured by:

1. Total number of all-cause ED visits;
2. Total number of all-cause 30-day ED revisits;
3. All-cause 30-day ED revisits rate;
4. Total number of all-cause discharges from IP;
5. Total number of all-cause 30-day IP readmissions;
6. All-cause 30-day IP readmission rate.

Intermediate measures will be measured by:

1. Number and proportion of patients to have identified HRSN;
2. Number and proportion of patients to receive referrals for their HRSN;
3. Number and proportion of patients to receive community-based services to meet their HRSN;
4. Connection to housing resources;
5. Connection to employment resources;
6. Connection to food;
7. Patient-level average of Proportion of Days Covered (PDC) for pharmaceutical prescriptions.

A budget impact analysis will be conducted to estimate the short-term expenditure changes and financial consequences of THRIVE+ compared to THRIVE-Basic, focusing on direct costs of implementation (staffing, resources, eligible intervention population size, etc.).

Post-hoc measures:
1. Social determinants of health services or referrals (e.g., food pantry referrals, food pantry visits, utilities shut-off protection letters).

5 Study Design

Design overview

We are performing a pragmatic trial of systematically screening for HRSN and providing a tailored set of printed referral guides that direct patients to specific hospital and community-based resources ("THRIVE-Basic"), relative to adding to THRIVE-Basic the assistance of pharmacy liaison-patient navigator, who provides targeted navigation services aimed at connecting patients with appropriate community resources (THRIVE+), to reduce hospital admissions and emergency department visits. Assignment to the study conditions is at the individual patient level. We will include adult patients age 18-64 receiving primary care in the General Internal Medicine Practice of Boston Medical Center who are members of the Boston Medical Center Medicaid ACO, and whose healthcare utilization ranks in the top 3-10% of the ACO membership. Because it is not practicable to obtain informed consent on all participants enrolled in the clinical program, we are requesting a waiver of informed consent for participants in both study arms. We are also requesting to obtain a waiver of HIPAA authorization. This will be justified in this protocol.

Setting and participants

We will conduct the study at the adult primary care practice at Boston Medical Center, the largest safety-net hospital in New England. We will pilot-test our intervention with two PCPs at Boston Medical Center. To be eligible, participants must 1) be age 18-64; 2) be within the 3rd to 10th percentile for healthcare utilization and cost among BACO membership (participants will therefore all be members of BACO) at the time of enrollment into the clinical program; 3) attend a primary care visit with a PCP (nurse practitioner or physician) in a GIM clinic at BMC. We will exclude patients who are currently receiving services from the BACO complex care management program, which includes support from a registered nurse, pharmacist, and community wellness advocate. Such patients may have been within the top two percentile for healthcare utilization and cost when they first enrolled in the complex care management program. However, over time their utilization and cost may have dropped to within the 3rd to 10th percentile. The Boston ACO identifies its high and intermediate risk populations through a statistical algorithm developed within its own information technology analytics group.

Follow-up data collection will occur over the course of 12 months, with repeated collection of primary, secondary, and intermediate outcome measures monthly among the THRIVE+ study arm participants to satisfy requirements of our funder.

6 Potential Risks and Benefits

6.1 Risks

Risks in this study are minimized, as this is a minimal risk trial. Potential risks are as follows:

1. Participants may feel uncomfortable being asked questions about their medication needs and HRSN. Some participants may not wish to speak to the pharmacy liaison-patient navigator about their needs

2. Participants may feel uncomfortable engaging in motivational interviewing (MI). MI is a counseling method to be delivered by the intervention patient navigators that encourages patients to discuss their unmet needs related to health outcomes and healthcare utilization (food and housing insecurity; need for transportation; trouble paying for heat, electric, or medications; need for employment or education; etc.). MI is a patient-centered way to encourage the subject to identify unmet needs and adopt behavior change that will promote engagement with resources and services to mitigate or alleviate HRSN.

3. Although we will make every effort to protect patient confidentiality, there is the possible risk of a breach of confidentiality; however, this study represents no more than minimal risk and the investigators anticipate the risk being no more than the risk under normal operations of accessing care in the health system.

Procedures to minimize risks include:

1. Participants will be made aware at the time of providing verbal agreement to participate in the clinical program that participation is strictly voluntary. If the patient gives verbal agreement to participate in the clinical program, they are under no obligation to engage in MI or patient

navigation. Refusal to answer a question will not affect their medical care and will not keep them from participating in the intervention.

2. Although we will make every effort to store data in a secure and confidential manner in concordance with IRB approval, there is the possible risk of a breach of confidentiality. If such a breach is determined to have happened, we will:
    a. Contact the BU/BMC IRB and BMC HIPAA Privacy Officer;
    b. Work to identify what happened and understand why;
    c. Develop a plan to rectify the situation and to contact all study participants whom the breach affected to explain what happened and who had access to the data; and
    d. Carry out the plan by contacting all study participants.

6.2 Potential Benefits

The participants involved in the intervention study arm stand a reasonable chance of benefiting, as the intervention components (screening-and-referral, motivational interviewing, and patient navigation) are all evidence-based.

The THRIVE-Basic model, already current practice in the hospital, is an adaptation of an evidence-based HRSN screening and referral program shown to increase vulnerable patients' receipt of community resources for HRSN.

The THRIVE+ intervention's underlying rationale is based on the tenets of patient navigation that reducing logistical and psychological barriers to services is an efficient and effective mechanism to increase engagement with services. Patient navigation has been used successfully in other domains to increase engagement with services, including cancer and mental health care. Although not currently standard practice in the clinical Pharmacy Care Program, patient navigation is used as standard practice at Boston Medical Center and in GIM specifically, where clinic-embedded patient navigators currently assist patients in identifying and connecting with hospital and community resources.

Future BMC GIM patients stand the opportunity to benefit from the knowledge we gain from the study.

6.3 Analysis of Risks in Relation to Benefits

The potential benefits of this comparative effectiveness study outweigh its risks. Both study arms (THRIVE-Basic usual care control arm and THRIVE+ intervention arm) are minimal risk interventions.

A focus on social determinants of health within the delivery of medical care offers promise for improving health outcomes and reducing the total cost of care – particularly among low-income populations.

Patients with higher medical complexity and more intensive acute health care utilization patterns often face a disproportionate share of HRSN as well as substantial barriers to access resources and services to

address those needs. Determining effective strategies to connect patients to resources that minimize, or eliminate, burden from HRSN —particularly strategies accessible to high-risk populations who share a disproportionate burden of HRSN—represents an opportunity not only to help the BACO patient population but also to identify cost-effective interventions and opportunities to reduce healthcare utilization.

Addressing HRSN of patients has substantial benefits for the ACO and is critical to advancing the ACO goals of improving quality of care and patient outcomes; reducing avoidable utilization; and providing care at the lowest possible cost. If successful, this project will allow for a scalable and sustainable intervention among a high-risk population.

7 Study Subject Selection

7.1 Subject Inclusion Criteria

To be eligible to participate in the study, an individual must meet all of the following criteria:
1. Age 18-64 years;
2. Be within the 3rd to 10th percentile for healthcare utilization and cost among BACO membership at the time of enrollment into the clinical program; and
3. Attend a primary care visit with a PCP (nurse practitioner or physician) in a BMC GIM clinic.

7.2 Subject Exclusion Criteria

An individual who meets any of the following criteria will be excluded from participation in this study:
1. Patients who are receiving services from the BACO complex care management program.

8 Study Intervention

We are comparing two usual care models in this comparative effectiveness trial: screening-and-referral (THRIVE-Basic) and enhanced screening-and-referral with patient navigation services and motivational interviewing (THRIVE+).

Current Clinical Practices: THRIVE-Basic and Pharmacy Care Program (usual care control arm)
The control arm for this comparative effectiveness trial is current standard of care in BMC GIM clinics.

Participants in the usual care control arm will receive (1) the paper THRIVE-Basic screener, the usual care low-touch screening and referral model and also receive (2) the services of a pharmacy liaison, as is current clinical practice in the GIM clinics.

1. The **THRIVE-Basic screening** tool includes eight HRSN domains: homelessness, housing insecurity, food insecurity, inability to afford medications, access to transportation for medical appointments, utilities, childcare, care for an elderly or disabled person, employment, and educational needs. Each screening domain is linked to an equivalent ICD-10 code to capture

these data in our EMR, and relevant paper resource referral guides are generated from the ICD-10 codes to provide contact information for local community and hospital resources to help patients address identified unmet social needs. Patients are engaged by asking not only if these domains represent challenges to them, but also if they want help connecting to resources to address these challenges. All documents are available in English, Spanish, Haitian Creole, and Portuguese.

Currently, all patients in BMC primary care clinics are eligible to receive the THRIVE-Basic screening at every primary care visit. However, not every patient completes the THRIVE screener at every visit due to staffing shortages, missed opportunities, and patients who decline to be screened. Patients who are screened complete a paper screener, which consists of two parts: part one screens patients for HRSN in the eight domains. Part two asks patients to indicate the resources they want help accessing across the eight domains. Once the patient is in an exam room, a medical assistant enters the screening results into the patients' electronic health record. Patients who request resources in one or more of the domains receive paper guides that describe available hospital and community resources to address the specific domain(s) they indicated. If patients need assistance accessing these resources, they may speak to a clinic-based patient navigator who supports the patients of approximately 12 part-time PCPs and who is also tasked with handling referrals to specialty services. The clinic-based navigator has access to the THRIVE directory to identify resources. The THRIVE directory is a web-based platform and allows for direct communication between community-based organizations and the clinic-based navigator for the purposes of follow-up.

2. Patients identified in the top 3-10% of BACO service utilization will also receive the **Pharmacy Care Program delivered by a pharmacy liaison**, as is current clinical practice in the GIM clinics. Patients are targeted for enrollment into this clinical program prior to or following their clinic visit. Within two weeks of verbal agreement to enrollment in the clinical program, the pharmacy liaison will conduct an initial intake assessment with the participant via telephone. During the assessment, the pharmacy liaison will review the participant's medication list, assess gaps in obtaining refills, identify barriers to medication adherence, review the participant's engagement in medical care, and develop an action plan with the participant. Action plans focus on strategies to increase medication adherence and engagement in care. The pharmacy liaison will also link the participant to a clinical pharmacist, when appropriate, and will encourage participants to transfer their prescriptions to one of the hospital's outpatient pharmacies. By having the participant transfer their prescriptions, the pharmacy liaison can better assist the participant with prescription management. After the initial intake is completed, the pharmacy liaison will call participants monthly (or meet with them prior to or following scheduled appointments) to confirm medication adherence and address any new barriers to medication adherence or engagement in medical care. . Pharmacy liaisons will review the most recent THRIVE screening results, and discuss any positive results. If the patient requests resources, the pharmacy liaisons will send an electronic message connect the patient to the clinic-based navigator, either in person or by way of an electronic message. The liaisons may also address health-related social needs if they are brought up by the patient and the liaison feels the needs

may impact the patient's ability to access or adhere to medications or access medical care. The pharmacy liaison in the control arm will does not systematically initiate screening for health-related social needs.

THRIVE+ (intervention arm)

Participants randomized to the intervention study arm will receive the usual care THRIVE-Basic paper screening and Pharmacy Care Program services (just as in the usual care THRIVE-Basic control arm, described above). However, the pharmacy liaison in the THRIVE+ arm will receive additional training to serve as a patient navigator and provide motivational interviewing in order to connect patients with community and hospital resources to address HRSN. Training will include acquisition of patient navigation and motivational interviewing skills through didactics, demonstrations, shadowing, role-plays, reading assignments, and video. The pharmacy liaison-patient navigators will practice the intervention protocol with "practice" participants. Training will also include information about best practices in screening for HRSN (e.g. "normalizing" the process, responding to participants' needs in an empathic and non-judgmental manner). During the intervention, a social worker with experience in motivational interviewing and connecting patients with HRSN to resources will meet every other week with the pharmacy liaison-patient navigators to discuss challenging cases.

The pharmacy liaison-patient navigators will spend the first 30-45 days after participant enrollment in the clinical program helping participants overcome any HRSN identified, above and beyond those brought up by the patient or barriers identified as impacting medication access and adherence. If the participant has not received screening for HRSN within the past three months, the pharmacy liaison-patient navigator will administer the THRIVE screener. For those participants with identified HRSN, the pharmacy liaison-patient navigator will perform a comprehensive assessment and will use the THRIVE directory and other resources to identify resources. The pharmacy liaison-patient navigators may use motivational interviewing skills to address domains in which participants screen positive but do not indicate they want resources. The pharmacy liaison-patient navigators will also provide patient education; assist with appointment-making and reminders; and offer social support, transportation, interpreter and child care services to ensure participants connect with resources to mitigate HRSN.

The pharmacy liaison-patient navigators will partner with a community organization, Action for Boston Community Development (ABCD). A dedicated ABCD staff member will help connect participants receiving THRIVE+ to ABCD community-based programs, services, and resources at ABCD's decentralized neighborhood sites and centralized departments. Resources include assistance with childcare, employment, utilities, and housing. The pharmacy liaison-patient navigators will initially assess patient needs, formulate recommendations for resources to address HRSN, and interface directly with the dedicated ABCD staff member to coordinate receipt of resources for their patients. Although BMC has a longstanding relationship with ABCD, prior to THRIVE+ there was no designated individual at ABCD to help connect BMC participants to its services, either directly or through their network of affiliations. One 0.5 FTE ABCD employee will be supported to help connect the THRIVE+ participants to services. After the initial 30-45-day intervention period, the pharmacy liaison-patient navigators will contact

participants monthly, re-administering the THRIVE screener every three months to assess for new or unmet HRSN needs until 1 year following enrollment in the clinical program.

9 Study Procedures

See the Appendix for the schedule of events.

We describe all study procedures, including duration, assignment to study arms, screening, intervention, and follow-up below.

Total Study Duration
We estimate that the study will take place from April 2019 (or when we receive IRB approval) to June 2021, including a 2-month pilot period (April – May 2019), 16 months of full study implementation (June 2019 – September 2020), and 12 months of post-enrollment in the clinical program follow-up (October 2020 – June 2021).

Subject Participation Duration
The duration of subject participation will be 12 months.

Screening Procedures for the Clinical Program – CURRENT STANDARD OF CARE IN BMC GIM
The first stage of screening for enrollment the clinical program is already being implemented as standard of care in BMC GIM clinics to identify patients in the 3 – 10 % risk tier of ACO membership for the pharmacy liaisons to target for enrollment in the clinical program. Pharmacy liaisons receive weekly reports from hospital clinical operations staff to identify patients in the 3rd to 10th percentile risk tier of ACO membership. The liaisons review each identified intermediate risk patient's medical record for study eligibility criteria. The pharmacy liaisons will not enroll patients into the clinical program who are identified as already receiving services from a higher-intensity complex care management program that targets patients with the highest level of health care utilization (top 1-2%). The pharmacy liaisons then review the GIM clinic schedule to identify eligible patients who are scheduled for clinic visits in one of the six GIM primary care clinics to target for enrollment in the clinical Pharmacy Care Program.

In all GIM primary care clinics, the second stage of screening for enrollment in the clinical program also occurs as part of existing clinic practice. After a patient checks in for an appointment, while waiting to be called into an exam room, they will be screened for unmet HRSN using the THRIVE-Basic screening tool. Upon completing the screen and entering the exam room, a medical assistant will enter the screening results into the EHR. Patients who request resources in one or more of the domains receive THRIVE-Basic paper guides that describe available hospital and community resources to address the specific domain(s) they indicated.

In order to avoid disrupting patient flow, all patients eligible for enrollment in the clinical program are approached for enrollment into the clinical program prior to or following their clinic visit, as is current practice. Upon the liaison receiving verbal agreement from the patient to participate in the clinical program, the liaison arranges with the patient a time to administer the pharmacy intake questionnaire via telephone within two weeks, as is standard practice in the Pharmacy Care Program. The liaison will

begin an initial assessment of the patient's prescription refill history in preparation for conducting the pharmacy intake assessment with the patient via phone.

<u>Assignment to Study Arm Procedures – THIS IS PART 1 OF THE RESEARCH PROVISIONS</u>

All study patients. For the research study, assignment to the study arms will center around these existing Pharmacy Care Program clinical program enrollment activities, which are a part of the current standard of care within GIM. The control arm pharmacy liaison will approach patients whose medical record number ends in an even number for enrollment in the clinical program, as they currently do as usual care; the intervention arm pharmacy liaison-patient navigator will approach patients whose medical record number ends in an odd number for enrollment in the clinical program. Patients with even medical record numbers who provide verbal agreement to the pharmacy liaison enroll in the clinical program will be assigned to the usual care control arm; patients with odd medical record numbers who provide verbal agreement to the pharmacy liaison-patient navigator to enroll in the clinical program will be assigned to the THRIVE+ intervention arm.

The intervention arm pharmacy liaison-patient navigator will describe their role and services offered by the THRIVE+ intervention (including the Pharmacy Care Program pharmacy services). Per usual clinical processes, the pharmacy liaisons do not use a script; they tailor the enrollment into the clinical program conversation to each individual patient. Pharmacy liaison-patient navigator will explain that BMC is implementing programs to assist patients with accessing medications and mitigating any barriers to accessing health and social services or supports. Pharmacy liaison-patient navigator will state that enrollment in the clinical program is completely voluntary.

If a patient declines or is not interested in the services offered by the pharmacy liaison-patient navigator, the navigator will thank the patient for their time.

<u>Intervention Procedures</u>

Usual Care Control Arm. *The control arm for this comparative effectiveness trial is current standard of care in BMC GIM clinics.* Participants receiving usual care in BMC GIM clinics will serve as the control arm for this study. Patients in the usual care control arm will receive THRIVE-Basic, as described in Section 8 of this protocol, and Pharmacy Care Program services delivered by a pharmacy liaison. These patients may also connect with a GIM clinic-based patient navigator (a separate individual from the pharmacy liaisons embedded within the GIM clinic). Participants will be followed for a total of 12 months.

Intervention Arm. At its core, this study is assessing whether a single intervention provider (i.e., having the intervention arm pharmacy liaison-patient navigator as a single individual that deploys both pharmacy services and patient navigation services as well as systematically screens for HRSN) is superior to multiple touches by separate, and often disparate, intervention providers (as in the usual care control arm). Participants in the study intervention study arm will receive the usual care THRIVE-Basic paper

screening and pharmacy program services (exactly as in the THRIVE-Basic usual care control arm). However, the patient navigator (a pharmacy liaison with additional training in patient navigation and motivational interviewing) will also systematically screen for HRSN and connect participants with resources at our community partner, ABCD. All intervention sessions (telephonic or in-person) will take place in private areas. Participants will be given options to maintain confidentiality during contact. For example, participants will be asked if it is okay to leave a telephone message and what is okay to say during the message from clinical staff to maintain confidentiality.

Following enrollment in the clinical program and assignment to study arm, study participants will be followed for a total of 12 months. Clinical program follow-ups will occur via monthly touches with the patient for 12 months (usually via phone or in-person if arranged at a time when the patient is in clinic) exactly as is currently done in the clinical program. Clinical staff (the liaisons) will use the preferred methods of contact indicated by the subject at enrollment in the clinical program and/or during completion of the intake assessment (i.e., phone calls, texts, email, etc.).

Research Follow-Up Procedures – THIS IS PART 2 OF THE RESEARCH PROVISIONS

We will assess outcome measures monthly for patients assigned to the THRIVE+ intervention arm, in order to satisfy a requirement of our funder. We will assess all outcome measures for all study participants at 6 months post-enrollment in the clinical program – again, to satisfy a requirement of our funder – and at 12 months post-enrollment in the clinical program.

As part of the research provisions, pharmacy liaisons-patient navigators will systematically screen patients assigned to the intervention arm with the THRIVE screener every three months to assess for new or unmet HRSN. These re-screens will take place during the monthly follow-up touches that are part of the existing clinical program.

We will analyze data collected via routine clinical care to ascertain primary and secondary study outcomes. There are three data sources for data collected as part of routine care:

1. BMC Clinical Data Warehouse
2. BMC HealthNet Claims Data
3. Pharmacy Care Program client tracking system

In addition, we will analyze data collected for research purposes. There is one data source for data collected for research purposes outside of routine clinical care:

1. Connection to resources at Action for Boston Community Development (ABCD), Inc.

To assess receipt of services our community partner for this study, ABCD, will check how many of the intervention arm study participants used services at ABCD to help address HRSN. ABCD will provide BMC with data to confirm patient receipt of services, the type of service received, and the date of receipt.

611 Table 1. Study Outcomes

Outcome measure	Data Source	Data collected as part of Routine Care or for Research?	Measurement Time Points (Post-Enrollment)
PRIMARY OUTCOME			
Acute healthcare utilization composite measure (hospital admissions and ED visits)	BMC HealthNet Plan claims data<br><br>BMC Clinical Data Warehouse	Routine Care	Monthly – for THRIVE+ intervention arm participants, only (funder requirement)<br><br>6 Months – all study participants (funder requirement)<br><br>12 Months – all study participants
SECONDARY OUTCOMES			
Total number of all-cause ED visits	BMC HealthNet Plan claims data<br><br>BMC Clinical Data Warehouse	Routine Care	Monthly – for THRIVE+ intervention arm participants, only (funder requirement)<br><br>6 Months – all study participants (funder requirement)<br><br>12 Months – all study participants
Total number of all-cause 30-day ED revisits	BMC HealthNet Plan claims data<br><br>BMC Clinical Data Warehouse	Routine Care	Monthly – for THRIVE+ intervention arm participants, only (funder requirement)<br><br>6 Months – all study participants (funder requirement)<br><br>12 Months – all study participants
All-cause 30-day ED revisit rate	BMC HealthNet Plan claims data<br><br>BMC Clinical Data Warehouse	Routine Care	Monthly – for THRIVE+ intervention arm participants, only (funder requirement)<br><br>6 Months – all study participants (funder requirement)<br><br>12 Months – all study participants
Total number of all-cause discharges from IP	BMC HealthNet Plan claims data<br><br>BMC Clinical Data Warehouse	Routine Care	Monthly – for THRIVE+ intervention arm participants, only (funder requirement)<br><br>6 Months – all study participants (funder requirement)<br><br>12 Months – all study participants
Total number of all-cause 30-day	BMC HealthNet Plan claims data	Routine Care	Monthly – for THRIVE+ intervention arm participants, only (funder requirement)

IP readmissions	BMC Clinical Data Warehouse		6 Months – all study participants (funder requirement)<br><br>12 Months – all study participants
All-cause 30-day IP readmission rate	BMC HealthNet Plan claims data<br><br>BMC Clinical Data Warehouse	Routine Care	Monthly – for THRIVE+ intervention arm participants, only (funder requirement)<br><br>6 Months – all study participants (funder requirement)<br><br>12 Months – all study participants
Intervention-level budget impact analysis	BMC HealthNet Plan claims data<br><br>Direct intervention implementation costs (staffing, resources, eligible intervention population size, etc.).	Routine Care	Study end
INTERMEDIATE OUTCOMES			
Patients to have identified HRSN	BMC Clinical Data Warehouse (THRIVE-Basic screening results)	Routine Care	Quarterly – All enrolled patients
Patients to receive referrals for their HRSN	BMC Clinical Data Warehouse (THRIVE-Basic paper referral guides provided)	Routine Care	Quarterly – All enrolled patients
Patients to receive services to meet their HRSN	Action for Boston Community Development data	Research	Quarterly – All enrolled patients
Connection to housing resources	Action for Boston Community Development data	Research	Quarterly – All enrolled patients
Connection to employment resources	Action for Boston Community Development data	Research	Quarterly – All enrolled patients
Connection to food	Action for Boston Community Development data	Research	Quarterly – All enrolled patients
Patient-level average of Proportion of Days Covered	BMC HealthNet Plan claims data	Routine Care	Quarterly – All enrolled patients

(PDC) for pharmaceutical prescriptions			

Table 2. Post-Hoc Measures

Post-hoc measure	Data Source	Data collected as part of Routine Care or for Research?	Measurement Time Points (Post-Enrollment)
PRIMARY OUTCOME			
Social determinants of health referrals and services	BMC Clinical Data Warehouse	Routine Care	12 Months – all study participants

10 Assessment of Safety and Data Safety Monitoring Plan (DSMP)

This study is not greater than minimal risk. For the purposes of this study:

1. An **Unanticipated Problem (UP)** will be defined as an event, experience or outcome that meets all three of the following criteria:
    a. Is unexpected; and
    b. Is related or possibly related to participation in the research; and
    c. Suggests that the research places subjects or others at a greater risk of harm (including physical, psychological, economic, or social harm) than was previously known or recognized.
2. An **Adverse Event (AE)** will be defined as any unanticipated problem or issue regarding confidentiality.

As this study is minimal risk, health-related AEs and Serious Adverse Events (SAEs) are not expected in this minimal risk study and therefore will not be measured.

Unanticipated Problems, Adverse Events, and protocol deviations will be reported to the IRB as required by IRB policies (http://www.bumc.bu.edu/ohra/hrpp-policies/hrpp-policies-procedures/#6.6.3).

The study monitor will be the Principal Investigator at Boston Medical Center/BU Medical Campus, and she will report all Adverse Events and Unanticipated Problems to the IRB in compliance with IRB policy, Federal/State regulations, and sponsor requirements (as applicable).

11 Data Handling and Record Keeping

11.1 Confidentiality

All participants will be assigned a unique study code. We will maintain participant confidentiality in the following ways:

1. The cross-walk that links study codes with identifying information will be kept in a secure network location and in a REDCap database, a database intended for large research studies which can securely manage HIPAA-sensitive data. There will be 2 separate REDCap databases: one solely for identifying information (the mastercode file, including subject name, medical record number, and code) and one solely for study data.
2. Each participant will receive a unique identification number; research data collection and data entry forms will be electronic and identified only with this number. Any hard copy materials will be kept in a locked file in the PI's locked office.
3. Computer systems will be password protected and accessible only to research staff needing the information for follow-up and monitoring purposes.
4. An important issue in web-based data capture is the potential for interception of information posted electronically, and interference with scripts and data stored on the server. To protect against security breaches, data will be electronically encrypted using secure socket layering so that only the intended recipient can decode. All laptops will be secured with password logins so data will be inaccessible in the event a unit is lost or stolen. Files stored on BUMC servers will be protected by electronic 'firewalls' that restrict access to designated users.

11.2 Source Documents

Source data/documents needed for this study that are part of the participant's standard of care treatment outside of the research include: (1) THRIVE-Basic screening results; (2) BMC HealthNet Plan claims data; (3) BMC EPIC clinical warehouse data; and (4) Pharmacy Care Program client tracking system.

1. THRIVE-Basic responses will be indicated by participants physically (on paper), and a medical assistant will transcribe the responses (electronically) into the participants' EMR. These data are collected in the routine course of clinical care.

2. We will analyze claims data to evaluate the primary healthcare utilization outcomes (overall hospital admissions; all-cause hospital readmissions; overall ED visits; ED revisits) and for the budget impact analysis.

3. Study outcomes and demographic variables will be obtained from de-identified patient-level data from the BMC clinical data warehouse. These data are collected in the routine course of clinical care.

4. Data will be obtained directly from participants by the clinical staff (the control pharmacy liaisons and the intervention pharmacy liaison-patient navigators). These data are also collected in the routine course of clinical care. Data generated by the methods described in the protocol

(including the Pharmacy Care Program Intake Assessment) will be recorded in the participants' medical records and/or the Pharmacy Care Program client tracking system.

11.3 Case Report Forms

This study does not require the use of case report forms for research purposes.

11.4 Study Records Retention

We will retain de-identified study records and the mastercode file 7 years after completion of the study, after which we will shred or permanently delete such documentation.

12 Statistical Plan

12.1 Study Hypotheses

We hypothesize THRIVE+ will increase connection to, and receipt of, resources to reduce the burden of HRSN, thereby reducing avoidable acute healthcare utilization, among intermediate risk BACO patients. This hypothesis is based on the evidence that the disproportionate utilization of the top 3-10% risk tier among BACO membership is driven in large part by social risk. We hypothesize that optimally addressing patients' HRSN will better positioned them to manage chronic conditions and adhere to preventive care plans, thereby reducing the likelihood they will use the emergency department or require inpatient care.

The null hypothesis is THRIVE+ does not reduce acute healthcare utilization (IP and ED visits) among intermediate risk BACO patients over the 12 month follow-up period.

The alternative hypothesis is THRIVE+ does reduce acute healthcare utilization (IP and ED) among intermediate risk BACO patients over the 12 month follow-up period.

12.2 Sample Size Determination

Sample Size. We derived the sample size that is needed to detect an overall difference which is of clinical significance and with potential cost-saving implications. We have powered our study to detect the primary outcome (10% relative reduction in overall hospital admissions and overall ED visits) at 12 months. We estimate 1,500 BACO members are eligible intermediate risk patients. We know from existing institutional data that these intermediate risk BACO members experience an average of 4.3 inpatient admission over 12 months, and 10.2 ED visits over 12 months. With these baseline data, we estimate that a total sample size of 364 patients (N=182 in each arm) is adequate to detect a 10% reduction in total ED plus hospital visits with statistical power of 0.90 and two-sided type I error rate of 0.05, accounting for 80% retention over a 12-month study period.

12.3 Statistical Methods

Study consort diagram.

Data Analysis Plan. Our primary outcome is the percentage change in all-cause hospital admissions and all-cause emergency department visits (a composite outcome) in the 12 months following enrollment. Secondary outcomes include total number of all-cause ED visits; all-cause 30-day ED revisits rate; total number of all-cause discharges from IP; all-cause 30-day IP readmission rate. Intermediate measures will be measured by: number and proportion of patients to have identified HRSN; number and proportion of patients to receive referrals for their HRSN; number and proportion of patients to receive community-based services to meet their HRSN; connection to housing resources; connection to employment resources; connection to food; and patient-level average of Proportion of Days Covered (PDC) for pharmaceutical prescriptions.

We will also collect data on demographics (age, gender, race, ethnicity, and language), insurance coverage (proportion of days covered from claims data), and on potential confounding variables, such as medical comorbidity as measured by the Elixhauser score), mental health and substance use diagnoses, THRIVE screening results, and medication adherence over 12 months using a measure of proportion of days covered based on pharmacy claims data.

We will first examine descriptive statistics of all variables. For categorical variables, we will report proportions, and for continuous variables, we will report means, standard deviations, and ranges. We will assess the randomness of treatment assignment according to odd versus even patient medical record number by comparing patient characteristics by treatment status. In order to determine if the THRIVE+ condition achieves a greater reduction (or smaller increase) in all-cause hospital admissions and all-cause emergency department visits (our composite primary outcome) in the 12 months following enrollment compared to the THRIVE-Basic condition, we will compare groups on their average number of visits in the 12 months post-intervention period controlling for potential baseline differences and any covariates (e.g., age, gender) determined *a priori* as important predictors of healthcare utilization. We will note differences that are statistically significant at the alpha < 0.05 level. We will assess the distribution of the outcome variables, diagnose error terms in residual analysis, and use appropriate link functions (e.g., normal link function for continuous outcomes with normal distribution) to construct linear regression models to assess the treatment effect. We will further assess any potential collinearity among covariates by evaluating variance inflation factors and correlation matrices. The primary analyses will be based on intent-to-treat. Because participants may receive varying “doses” of the THRIVE+ intervention, we will also analyze the intervention effect according to whether participants received a “minimum dose” of the intervention. In order to assess whether the presence of comorbid mental health and substance use diagnoses, race, and language may modify the treatment effect, we will evaluate interaction terms between these pre-determined potential moderators and the treatment variable in the overall sample, and further conduct stratified analyses among these potential modifiers to assess the robustness of any differential effects if detected above.

We will report our results according to CONSORT guidelines.

De-identified data for inclusion in consort diagram. We will review de-identified to develop our consort flow diagram depicting the process through which patients were approached, recruited, enrolled into the clinical program, and attributed to each of the two study comparison groups. Specifically, we will explore reasons for drop-off between patients approached for enrollment in the clinical program and patients who were ultimately attributed to the trial. We will review de-identified data for patients who drop-off: (1) BMC pharmacy intervention provider name (which indicates study arm) and (2) the reason for not being enrolled in clinical program. These are source data collected by clinical Pharmacy programs in the course of routine care within the program's client tracking system.

13 Ethics/Protection of Human Subjects

This study is to be conducted according to applicable US federal regulations and institutional policies (which are based in federal regulations, guidance, and ICH Good Clinical Practice guidelines).

This protocol and any amendments will be submitted to the Boston Medical Center and Boston University Medical Campus IRB, for formal approval of the study conduct. The decision of the IRB concerning the conduct of the study will be made in writing to the investigator. A copy of the initial IRB approval letter will be provided to the sponsor before commencement of this study.

We believe that this study meets all four requirements of the Code of Federal Regulations (45 CFR 46.116 (d)) regarding waiver of consent:

1. The investigators consider this study to be **no greater than minimal risk** to the subjects. All study components (THRIVE screening and referral; patient navigation) are current standard of care at Boston Medical Center. For patients in the usual care control arm, the study is solely collecting data as subjects in the control group will have care as usual (THRIVE-Basic and Pharmacy Care Program services). Subjects in the intervention arm (THRIVE+) are likely to have higher level of attentiveness to their HRSN than without the study. The THRIVE+ intervention's underlying rationale is based on the tenets of patient navigation. Although not currently standard practice in the clinical Pharmacy Care Program, patient navigation is used as standard practice at Boston Medical Center and in GIM specifically, where clinic-embedded patient navigators currently assist patients in identifying and connecting with hospital and community resources. At its core, this study is assessing whether streamlining service delivery (i.e., having the intervention arm pharmacy liaison-patient navigator as a single individual that deploys both pharmacy services and patient navigation services as well as systematically screens for HRSN) is superior to multiple touches by separate, and often disparate, resources (i.e., a pharmacy liaison and a separate clinic-embedded patient navigator each delivering different services not systematically linked to screening for HRSN). We have a very strong data security plan to prevent any data breach, further minimizing potential risks.

2. **Waiving the requirements for informed consent will not adversely affect the rights and welfare of study subjects**, given the potential for harm or discomforts are not greater than those encountered during usual care.

3. **The research cannot be practicably carried out without the waiver of informed consent or alteration of the consent process**. Because this is a real-world comparative effectiveness study comparing two standard-of-care interventions, we are studying whether changing systems of care affects patient outcomes. The services that will be delivered to both study arms (intervention and control) are standard of care; it is not experimental. Given this comparative effectiveness study of THRIVE-Basic vs. THRIVE+ exists within the context of a usual care program (Pharmacy Care Program), it would be impracticable to consent subjects to be in the research. The consent process would result in a study sample that is highly selected—not a real-world sample. In addition, patients will not be enrolled into the study by a member of the study staff; rather, clinical staff (the liaisons) will identify and enroll patients into the clinical program, as is standard practice in the Pharmacy Care Program. Assignment to study arms will be based on medical record number (even vs. odd). As articulated by McKinney et al,[20] we believe that a requirement to obtain informed consent would "impose a significant or even insurmountable barrier" to conducting this pragmatic clinical research trial, by hampering recruitment. Further, requiring informed consent has the "potential to harm future patients by depriving them and their healthcare providers of the evidence needed to guide care." We also believe that requiring informed consent would limit our trial's generalizability. The type of patient who has the time and motivation to undergo a long consent process may not be representative of the patient population receiving primary care at Boston Medical Center and in other safety-net settings. Finally, the interventions to be compared in our study convey benefit (and not risk) to patients, and do not require consent outside the research context. Dr. Lasser has conducted two prior clinical trials[10,11] similar to the present study (comparing two versions of standard care) where a waiver of informed consent was obtained and the studies produced important scientific knowledge that has influenced care delivery in primary care.

4. This study involves using identifiable information, as the investigators will need to prospectively follow data for all study subjects in order to assess study outcomes (acute health care utilization and accessing services for HRSN) over the course of the study. The **research cannot practicably be carried out without using such information** as we would be unable to ascertain study outcomes for both study arms.

5. Criterion 5 is **not applicable**, as dissemination of pertinent information to study subjects after the study is completed is not applicable to this minimum risk study where both study arms will receive standard practice of care services.

We believe the research cannot be practicably carried out without the waiver of HIPAA authorization because this is a real-world comparative effectiveness study comparing two standard-of-care interventions. We are studying whether changing systems of care affects patient outcomes. The investigators will need to prospectively follow data, including PHI, for all study subjects in order to assess study outcomes (acute health care utilization and accessing services for HRSN) up to 12 months post study enrollment. The services that will be delivered to both study arms (screening and referral; patient navigation) are standard of care; it is not experimental. Given this comparative effectiveness study of THRIVE-Basic vs. THRIVE+ exists within the context of a usual care clinical program (Pharmacy Care Program), it would be impracticable to obtain HIPAA authorization from subjects to be in the research. The process would result in a study sample that is highly selected—not a real-world sample. In

addition, patients will not be enrolled into the study by a member of the study staff; rather, clinical staff (the liaisons) will identify and enroll patients into the clinical program, as is standard practice in the Pharmacy Care Program. Patients will then be assigned to study arms by medical record number.

14 Literature References

1. Marmot MG, Wilkinson R. *The solid facts: social determinants of health.* Centre for Urban Health, World Health Organization; 1998.
2. Silverstein M, Conroy K, Sandel M. Screening for social determinants of health in pediatric primary care. *Pediatric annals.* 2008;37(11).
3. Silverstein M, Mack C, Reavis N, Koepsell TD, Gross GS, Grossman DC. Effect of a clinic-based referral system to head start: a randomized controlled trial. *JAMA.* 2004;292(8):968-971.
4. Machledt D. Addressing the Social Determinants of Health Through Medicaid Managed Care. *Issue brief (Commonwealth Fund).* 2017;2017:1-9.
5. Gifford K, Ellis E, Coulter Edwards B, et al. Medicaid moving ahead in uncertain times: Results from a 50-state medicaid budget survey for state fiscal years 2017 and 2018. *Kaiser Family Foundation. www. kff. org/medicaid/report/medicaid-moving-ahead-in-uncertain-times-results-from-a-50-state-medicaid-budget-survey-for-state-fiscal-years-2017-and-2018/. Accessed January.* 2017;17:2018.
6. Gottlieb LM, Wing H, Adler NE. A systematic review of interventions on patients' social and economic needs. *American journal of preventive medicine.* 2017;53(5):719-729.
7. Garg A, Toy S, Tripodis Y, Silverstein M, Freeman E. Addressing social determinants of health at well child care visits: a cluster RCT. *Pediatrics.* 2015;135(2):e296-e304.
8. Social IoMCotR, Domains B, Records MfEH. *Capturing social and behavioral domains and measures in electronic health records: phase 2.* National Academies Press; 2014.
9. Battaglia TA, Roloff K, Posner MA, Freund KM. Improving follow-up to abnormal breast cancer screening in an urban population: a patient navigation intervention. *Cancer: Interdisciplinary International Journal of the American Cancer Society.* 2007;109(S2):359-367.
10. Lasser KE, Quintiliani LM, Truong V, et al. Effect of patient navigation and financial incentives on smoking cessation among primary care patients at an urban safety-net hospital: a randomized clinical trial. *JAMA internal medicine.* 2017;177(12):1798-1807.
11. Lasser KE, Murillo J, Lisboa S, et al. Colorectal cancer screening among ethnically diverse, low-income patients: a randomized controlled trial. *Archives of internal medicine.* 2011;171(10):906-912.
12. Silverstein M, Diaz-Linhart Y, Grote N, Cadena L, Cabral H, Feinberg E. Patient navigation for depressed mothers in Head Start: A pilot study of intervention mechanism. *Journal of Community Psychology.* 2017;45(4):564-570.
13. Warning W, Wood J, Letcher A, Srouji N, Echterling C, Carpenter C. Working with the Super Utilizer Population: The Experience and Recommendations of Five Pennsylvania Programs. *Aligning Forces for Quality, The Highmark Foundation.* 2014.
14. Gunn C, Battaglia TA, Parker VA, et al. What makes patient navigation most effective: defining useful tasks and networks. *Journal of health care for the poor and underserved.* 2017;28(2):663-676.
15. Ramachandran A, Snyder FR, Katz ML, et al. Barriers to health care contribute to delays in follow-up among women with abnormal cancer screening: Data from the Patient Navigation Research Program. *Cancer.* 2015;121(22):4016-4024.
16. Freeman HP, Rodriguez RL. History and principles of patient navigation. *Cancer.* 2011;117(S15):3537-3540.
17. Diaz-Linhart Y, Silverstein M, Grote N, et al. Patient navigation for mothers with depression who have children in head start: a pilot study. *Social work in public health.* 2016;31(6):504-510.

18. Battaglia TA, Burhansstipanov L, Murrell SS, et al. Assessing the impact of patient navigation: prevention and early detection metrics. *Cancer.* 2011;117(S15):3551-3562.
19. Lasser KE, Kenst KS, Quintiliani LM, et al. Patient navigation to promote smoking cessation among low-income primary care patients: a pilot randomized controlled trial. *Journal of ethnicity in substance abuse.* 2013;12(4):374-390.
20. McKinney Jr RE, Beskow LM, Ford DE, et al. Use of altered informed consent in pragmatic clinical research. *Clinical Trials.* 2015;12(5):494-502.

15 Appendix

Schedule of Events

ATTACHMENT 1. Schedule of events/ data collection **FOR RESEARCH PROVISIONS**

		**Screening and Baseline Visit**		**1-2 weeks**	**6 Months**	**12 Months**	**Monthly** ***(intervention arm patients, only)***	**Quarterly**
		**Screener**	**Baseline**					
Screening* ***Part of the <u>clinical program</u>***	Identification as intermediate risk ACO member by statistical algorithm	X						
	Chart/EMR review for clinical program eligibility	X						
Enrollment* ***Part of the <u>clinical program</u>***	Verbal Consent		X					
	Pharmacy Intake questionnaire			X				
Intervention	Assignment to study arm by medical record number		X					
	THRIVE+ Screener							X
Assessments ***Part of the <u>clinical program</u>***	Follow-up assessments						X	
Data Collection	BMC Clinical Data Warehouse				X	X	X	X
	BMC HealthNet Claims Data				X	X	X	X
	Connection to resources at Action for ABCD							X

Title: A Pharmacy Liaison-Patient Navigation Intervention to Reduce Inpatient and Emergency Department Utilization among Primary Care Patients in a Medicaid Accountable Care Organization: A Pseudo-randomized Controlled Trial

Statistical Analysis Plan

Version 1.0

26 November, 2021

**Principal Investigator:**<br><br><br><br><br><br>**Methodologist and statistician:**	Karen E. Lasser, MD, MPH<br>Section of General Internal Medicine<br>Boston Medical Center<br>801 Massachusetts Ave, 6th Floor<br>Boston, MA 02118<br><br>Ziming Xuan, ScD<br>Department of Community Health Sciences<br>Boston University School of Public Health<br>Crosstown Building – CT 454<br>801 Massachusetts Avenue<br>Boston, MA 02118<br><br>**Data analyst:**<br>Na Wang, MA<br>Biostatistics and Epidemiology Data Analytics Center (BEDAC)<br>Boston University School of Public Health<br>85 East Newton Street, M921<br>Boston, MA 02118
**Sponsor:** Health Policy Commission; John Hancock	
**Rationale for pharmacy liaison with motivational interviewing training to provide targeted navigation services to connect patients to community services in order to reduce health care utilization**	Pharmacy liaison with motivational interviewing training may help patients prioritize unmet social needs (e.g., lack of food or housing) along with medication adherence and receipt of medical care and may lead to reduced utilization of medical services. A focus on health-related social needs within the delivery of medical services offers opportunities for improving health outcomes and reducing the cost of care, especially among low-income patients such as those served by safety-net hospitals. This intervention is compared to the standard of care in the general internal medicine practice where medical staff screen patients for health-related social needs.
**Inclusion and exclusion criteria**	As part of usual care, the Accountable Care Organization (ACO) identifies these two patient populations (e.g., 2nd percentile vs.

	within 3rd to 10th percentile of utilization) through an assessment of clinical data as well as claims data from a Medicaid payer common to all ACO beneficiaries.<br><br>Eligibility criteria for the program are: 1) age 18–64; 2) within the 3rd to 10th percentile for health care utilization and cost among Medicaid Accountable Care Organization (ACO) membership at the time of enrollment into the pharmacy program; and 3) attended a visit with a primary care provider (nurse practitioner or physician).<br><br>We exclude patients that are eligible for or are receiving services from a more intensive ACO care management program that targets patients within the 1st to 2nd percentile for health care utilization.
**Randomization and treatment procedures**	Patients are assigned to study conditions based on medical record number. The usual care pharmacy liaisons approach and recruit patients with an even medical record number, while the enhanced usual care pharmacy liaisons approach and recruit patients whose medical record number ends in an odd number.<br><br>Assignment to treatment condition is unblinded. However, the patients are not aware of assigned study arms.<br><br>**Procedures:**<br>**Usual Care Control Arm**<br>Medical assistants screen patients for health-related social needs. Patients who request resources receive printed language congruent paper guides to address the specific domain(s) indicated during their clinic visit. Pharmacy liaisons offer their services to the patients during the patient's scheduled primary care appointment. The pharmacy liaison in the usual care arm does not systematically screen for health-related social needs.<br><br>**Enhanced Usual Care Arm**<br>Patients assigned to the enhanced usual care study arm receive the screening and pharmacy program services described above. Additionally, they have more time to spend

	with each patient and receive training to serve as patient navigators with a specific focus on connecting patients with community and hospital resources to address health-related social needs. They also receive training in motivational interviewing. The pharmacy liaison-patient navigators spend the first 30–45 days after patient enrollment in the pharmacy program helping patients overcome any health-related social needs identified, beyond those identified as barriers to medication adherence. The pharmacy liaison-patient navigator also provides patient education; assists with scheduling appointments and reminders; and offers support to connect with internal and community resources to help patients mitigate health-related social needs.
**Duration of follow-up**	12 months
**Criteria for efficacy**	**Measures:**<br>We collect data on demographics (age, gender, race, ethnicity, and language), insurance coverage (proportion of days covered from claims data), and on potential confounding variables, such as medical comorbidity as measured by a validated comorbidity index (the Charlson Comorbidity Score), mental health and substance use diagnoses, health-related social needs screening results, and medication adherence using a measure of proportion of days covered based on pharmacy claims data.<br><br>**Primary end points:**<br>Our primary outcome is the percentage change in all-cause hospital admissions and all-cause emergency department visits (a composite outcome) in the 12 months following enrollment.<br><br>**Secondary end points:**<br>Secondary outcomes include all-cause hospital admissions and all-cause emergency department visits as separate outcomes, all-cause 30-day hospital readmissions, 30-day ED revisit rates, and connection to resources (referral to food pantry, visits to food pantry, utility shutoff protection letters).
**Statistical method**	We compare baseline characteristics of participants using two-sample t tests for continuous variables or Chi-square tests for

	categorical variables. Due to a potential high number of patients with zero-count visits (136 out of 364, 37%), we employ a zero-inflated negative binomial model, which is a two-part model that examines simultaneously the odds of any visit, and the rates of visits among those patients who have any health care utilization. We use Bayesian Information Criteria as the model goodness-of-fit measure to compare zero-inflated negative binomial model with other models for the rates outcomes, including over-dispersed Poisson model, and negative binomial model. As a sensitivity analysis, we also model only the binary outcome of any utilization using logit link function. In the adjusted regression models, we will control for any factors including baseline utilization that are found statistically significant between the two arms. We report odds ratios and 95% CI for binary outcome (e.g., any visit) and rate ratios for the outcomes of inpatient and ED visits. We also compare the secondary outcomes of health-related social needs (e.g., connected to community-based partner; received services through referral to community partner; referrals and visits to hospital food pantry; received utility shutoff protection letter) between the two arms and examine adjusted intervention effect using logistic regression with odds ratio and 95% CI.<br><br>The primary analyses will be based on intent-to-treat. Because participants may receive varying "doses" of the intervention, we will also analyze the intervention effect according to whether participants received a minimum dose of the intervention. If the pharmacy liaison-patient navigator documents in their first telephone assessment that the patient has received the THRIVE screening in the past three months, and rescreens the patient if they have not been screened in the past three months, we consider the patient to have received the minimum intervention dose.

2. Major amendments to the protocol

None

3. Analysis population

3.1 Flow diagram

Please see manuscript Figure.

3.2 Definition of the analysis population

Study recruitment took place from May 2019 through March 2020 at a large primary care internal medicine practice affiliated with an academic safety-net hospital in Boston, Massachusetts. Patients that qualified for the hospital's pharmacy care program were eligible for the study. Eligibility criteria for the program were: 1) age 18–64; 2) within the 3rd to 10th percentile for health care utilization and cost among Medicaid Accountable Care Organization (ACO) membership at the time of enrollment into the pharmacy program; and 3) attended a visit with a primary care provider (nurse practitioner or physician).

3.3 Sample size

We used internal health care utilization data from the institution for sample size calculation. Using a two-sided type-I error rate of 0.05, a sample size of 364 patients (N=182 in each arm) would achieve 90% power to detect a 10–percentage point reduction in our primary outcome of total emergency department visits plus inpatient hospital admissions.

4. Analysis principles

4.1 General principles for analysis of outcomes

The primary analyses are based on intent-to-treat. Because participants may receive varying "doses" of the intervention, we also analyze the intervention effect according to whether participants received a minimum dose of the intervention.

In order to assess whether the presence of comorbid mental health and substance use diagnoses, race, and language may modify the treatment effect, we will evaluate interaction terms between these pre-determined potential moderators and the treatment variable in the overall sample, and further conduct stratified analyses among these potential modifiers to assess the robustness of any differential effects due to effect modification.

4.2 Participants' characteristics at inclusion

Table 1 shows descriptive characteristics for the usual care and enhanced usual care groups. The usual care and enhanced usual care groups differed in the respective proportion who spoke English (78% vs. 87%), reported housing insecurity (9% vs. 13%), received a referral to the hospital food pantry in the past 12 months (14% vs. 25%), had at least one visit to hospital food pantry in past 12 months (12% vs. 20%) and had a diagnosis of PTSD (21% vs. 14%). The majority belonged to a racial/ethnic minority group, and 96% had received screening for health-related social needs in the past year. Approximately two-thirds of patients had significant medical comorbidity, and nearly half carried a diagnosis of depression.

4.3 Handling of missing or incoherent data

We assess the distribution of all variables and the extent of missingness. Outlier or incoherent data will be discussed and addressed by consensus among the investigative team. Given the nature of health care utilization recorded in the clinical data warehouse and claims database, it is not expected that the primary and secondary outcomes data would be missing.

4.4 Statistical software

We use SAS Version 9.4 to conduct all statistical analyses (SAS Institute Cary, NC).

5. Primary outcome analysis

5.1 Definitions

Our primary outcome is all-cause hospital admissions and all-cause emergency department visits (a composite outcome) in the 12 months following enrollment. We compare baseline characteristics of participants using two-sample t tests for continuous variables or Chi-square tests for categorical variables. We assess the distribution of the outcome variables, diagnose error terms in residual analysis, and use appropriate link functions (e.g., normal link function for continuous outcomes with normal distribution) to construct linear regression models to assess the treatment effect. We will further assess any potential collinearity among covariates by evaluating variance inflation factors and correlation matrices. In the case where there are excess zeros (e.g., no visits), we employ a zero-inflated negative binomial model, which is a two-part model that examines simultaneously the odds of any visit, and the rates of visits among those patients who had any health care utilization. In the adjusted regression models, we will control for any factors including baseline utilization that are found statistically significant between the two arms.

5.2 Amended definitions

None

5.3 Trial monitoring

5.3.1 Interim analyses

We will conduct a 6-month interim analysis to assess data quality, examine distribution of variables and potential incoherence, and conduct preliminary analyses to detect between-group difference in health care utilization at 6-month follow up.

5.3.2 Presentation of results

We examine descriptive statistics of all variables. For categorical variables, we will report proportions, and for continuous variables, we will report means, standard deviations, and ranges. We will assess the randomness of treatment assignment according to odd versus even patient medical record number by comparing patient characteristics by treatment status. In order to determine if the enhanced usual care intervention achieves a greater reduction (or smaller increase) in all-cause hospital admissions and all-cause emergency department visits (our composite primary outcome), respectively, in the 12 months following enrollment compared to the usual care condition, we report and compare groups on their average number of visits in the 12 months post-enrollment controlling for potential baseline differences and any covariates (e.g., age, gender) determined *a priori* as important predictors of healthcare utilization. We will note differences that are statistically significant at the alpha < 0.05 level. In the adjusted regression models, we control for any factors including baseline utilization that are found statistically significant between the two arms.

5.4 Calculation of the outcome

A composite measure is created by summing all-cause hospital admissions and all-cause emergency department visits (a composite outcome) in the 12 months following enrollment.

5.5 Subgroup analyses

In order to assess whether the presence of comorbid mental health and substance use diagnoses, race, and language may modify the treatment effect, we evaluate interaction terms between these pre-determined potential moderators and the treatment variable in the overall sample, and further conduct stratified analyses among these potential modifiers to assess the robustness of any differential effects if detected above. We conduct stratified analyses where we have adequate sample size (age, gender, race, language).

6. Secondary efficacy outcomes analysis

6.1 Definitions

Secondary outcomes include all-cause hospital admissions and all-cause emergency department visits as separate outcomes, all-cause 30-day hospital readmissions, 30-day ED revisit rates, and connection to resources (visits to food pantry, receipt of utility shutoff protection letter).

6.2 Methods for analysis

We will use similar methodology above to examine bivariate comparison and conduct adjusted analyses to examine the secondary outcomes.

7. Safety analysis

N/A, this study involved no more than minimal risk to subjects.
