# Supplementary material for: A Pharmacy Liaison–Patient Navigation Intervention to Reduce Inpatient and Emergency Department Utilization Among Primary Care Patients in a Medicaid Accountable Care Organization: A Nonrandomized Controlled Trial
Source: JAMA Netw Open. 2023 Jan 9;6(1):e2250004. doi: 10.1001/jamanetworkopen.2022.50004 (PMC9856667; doi:10.1001/jamanetworkopen.2022.50004)
Supplement: Supplement 2. — eTable 1. Analyses Restricted to Patients With Continuous ACO Enrollment During the Follow-up Period eTable 2. Analyses Comparing Patients in the Enhanced Pharmacy Care Group According to Receipt of the Minimum Intervention Dose [file jamanetwopen-e2250004-s002.pdf]

Supplementary Online Content

Buitron de la Vega P, Ashe EM, Xuan Z, et al. A pharmacy liaison–patient navigation intervention to reduce inpatient and emergency department utilization among primary care patients in a Medicaid accountable care organization: a nonrandomized controlled trial. *JAMA Netw Open*. 2023;6(1):e2250004. doi:10.1001/jamanetworkopen.2022.50004

eTable 1. Analyses Restricted to Patients With Continuous ACO Enrollment During the Follow-up Period

eTable 2. Analyses Comparing Patients in the Enhanced Pharmacy Care Group According to Receipt of the Minimum Intervention Dose

This supplementary material has been provided by the authors to give readers additional information about their work.

© 2023 Buitron de la Vega P et al. *JAMA Network Open*.

eTable 1. Analyses Restricted to Patients With Continuous ACO Enrollment During the Follow-up Period

	Follow-up				Adjusted Zero-Inflated Negative Binomial Model[a]			
	Enhanced Pharmacy Care (n=109)		Usual Pharmacy Care (n=108)		Zero-inflated model		Negative binomial model	
Variable	Number of patients with any hospital admission or emergency department visit n (%)	Mean number of hospital admissions or emergency department visits among patients with any visit (sd)	Number of patients with any hospital admission or emergency department visit n (%)	Mean number of hospital admissions or emergency department visits among patients with any visit (sd)	Odds ratio (95% CI)	p-value	IRR (95% CI)	p-value
All-cause inpatient hospital admissions and all-cause emergency department visits (a composite outcome) within the past year	64 (58.7)	4.5 (4.3)	71 (65.7)	3.7 (4.0)	0.41 (0.12, 1.45)	0.17	1.13 (0.80, 1.61)	0.49
All-cause emergency department visits within the past year	61 (55.9)	3.7 (3.4)	69 (63.9)	3.2 (3.3)	0.45 (0.14, 1.53)	0.20	1.09 (0.77, 1.54)	0.63
30-day emergency department revisits within the past year	35 (32.1)	2.8 (2.8)	33 (30.6)	2.9 (3.2)	1.21 (0.28, 5.34)	0.80	1.05 (0.54, 2.02)	0.89
All-cause inpatient hospital admissions within the past year	25 (22.4)	2.4 (1.9)	21 (19.4)	2.0 (1.2)	1.59 (0.35, 7.18)	0.55	0.93 (0.44, 1.98)	0.86
All-cause 30-day inpatient hospital readmissions within the past year[b]	11 (10.1)	2.3 (2.0)	10 (9.3)	1.3 (0.5)	0.40 (0.08, 2.11)	0.28	2.36 (0.84, 6.65)	0.10

[a] Zero-inflated negative binomial model adjusted for English, baseline housing insecurity, PTSD, referral to food pantry, visit to food pantry, and baseline outcome.

[b] The validity of the model fit is questionable in adjusted model due to convergence problem.

© 2023 Buitron de la Vega P et al. *JAMA Network Open*.

eTable 2. Analyses Comparing Patients in the Enhanced Pharmacy Care Group According to Receipt of the Minimum Intervention Dose[a]

	Follow up				Adjusted Zero-Inflated Negative Binomial Model[b]			
	Received Minimum Intervention Dose (n=105)		Did not received Minimum Intervention Dose (n=77)		Zero-inflated model		Negative binomial model	
Variable	Proportion of patients with any hospital admission or emergency department visit n (%)	Mean number of hospital admissions or emergency department visits among patients with any visit (sd)	Proportion of patients with any hospital admission or emergency department visit n (%)	Mean number of hospital admissions or emergency department visits among patients with any visit (sd)	Odds ratio (95% CI)	p-value	IRR (95% CI)	p-value
All-cause inpatient hospital admissions and all-cause emergency department visits (a composite outcome) within the past year	62 (59.0)	4.1 (3.9)	48 (62.3)	4.5 (4.8)	2.10 (0.57, 7.70)	0.87	0.97 (0.65, 1.43)	0.26
All-cause emergency department visits within the past year	59 (56.2)	3.6 (3.4)	47 (61.0)	3.7 (3.6)	3.24 (0.36, 29.12)	0.81	0.95 (0.64, 1.41)	0.29
30-day emergency department revisits within the past year	30 (28.6)	3.0 (3.1)	26 (33.8)	3.4 (3.5)	0.47 (0.12, 1.82)	0.12	1.65 (0.88, 3.08)	0.28
All-cause inpatient hospital admissions within the past year[c]	23 (21.9)	2.0 (1.0)	18 (23.4)	2.6(2.2)	1.87 (0.33, 10.40)	0.41	0.65 (0.23, 1.82)	0.48
All-cause 30-day inpatient hospital readmissions within the past year[c]	11 (10.5)	1.4 (0.7)	6 (7.8)	3.2 (2.5)	1.60 (0.18, 14.38)	0.56	1.48 (0.39, 5.60)	0.68

[a] If the pharmacy liaison-patient navigator documented during the intake assessment that the patient had received screening for health-related social needs in the past three months, and rescreened the patient if they had not been screened in the past three months, we considered the patient to have received the minimum intervention dose.
[b] Zero-inflated negative binomial model adjusted for English, baseline housing insecurity, PTSD, referral to food pantry, visit to food pantry, and baseline outcome.
[c] The validity of the model fit is questionable in adjusted model due to convergence problem.

© 2023 Buitron de la Vega P et al. *JAMA Network Open*.
