# Supplementary material for: A Pharmacy Liaison–Patient Navigation Intervention to Reduce Inpatient and Emergency Department Utilization Among Primary Care Patients in a Medicaid Accountable Care Organization: A Nonrandomized Controlled Trial
Source: JAMA Netw Open. 2023 Jan 9;6(1):e2250004. doi: 10.1001/jamanetworkopen.2022.50004 (PMC9856667; doi:10.1001/jamanetworkopen.2022.50004)
Supplement: Supplement 3. — Data Sharing Statement [file jamanetwopen-e2250004-s003.pdf]

Data Sharing Statement

Buitron de la Vega. A Pharmacy Liaison-Patient Navigation Intervention to Reduce Inpatient and Emergency Department Utilization Among Primary Care Patients in a Medicaid Accountable Care Organization. *JAMA Netw Open*. Published January 09, 2023. doi:10.1001/jamanetworkopen.2022.50004

Data
Data available: Yes
Data types: Deidentified participant data
How to access data: karen.lasser@bmc.org
When available: With publication

Supporting Documents
Document types: None

Additional Information
Who can access the data: researchers whose proposed use of the data has been approved
Types of analyses: any purpose
Mechanisms of data availability: without investigator support
